# Small area variations in low birth weight and small size of births in India

**DOI:** 10.1111/mcn.13369

**Published:** 2022-04-29

**Authors:** Md Juel Rana, Rockli Kim, Soohyeon Ko, Laxmi K. Dwivedi, K. S. James, Rakesh Sarwal, S. V. Subramanian

**Affiliations:** ^1^ International Institute for Population Sciences Mumbai Maharashtra India; ^2^ Division of Health Policy and Management, College of Health Science Korea University Seoul South Korea; ^3^ Interdisciplinary Program in Precision Public Health, Department of Public Health Sciences Graduate School of Korea University Seoul South Korea; ^4^ National Institution for Transforming India (NITI) Aayog, Government of India New Delhi India; ^5^ Harvard Center for Population and Development Studies Cambridge Massachusetts United States

**Keywords:** child public health, epidemiology, inequalities, low birth weight, low income countries, maternal public health

## Abstract

The states and districts are the primary focal points for policy formulation and programme intervention in India. The within‐districts variation of key health indicators is not well understood and consequently underemphasised. This study aims to partition geographic variation in low birthweight (LBW) and small birth size (SBS) in India and geovisualize the distribution of small area estimates. Applying a four‐level logistic regression model to the latest round of the National Family Health Survey (2015–2016) covering 640 districts within 36 states and union territories of India, the variance partitioning coefficient and precision‐weighted prevalence of LBW (<2.5 kg) and SBS (mother's self‐report) were estimated. For each outcome, the spatial distribution by districts of mean prevalence and small area variation (as measured by standard deviation) and the correlation between them were computed. Of the total valid sample, 17.6% (out of 193,345 children) had LBW and 12.4% (out of 253,213 children) had SBS. The small areas contributed the highest share of total geographic variance in LBW (52%) and SBS (78%). The variance of LBW attributed to small areas was unevenly distributed across the regions of India. While a strong correlation between district‐wide percent and within‐district standard deviation was identified in both LBW (*r* = 0.88) and SBS (*r* = 0.87), they were not necessarily concentrated in the aspirational districts. We find the necessity of precise policy attention specifically to the small areas in the districts of India with a high prevalence of LBW and SBS in programme formulation and intervention that may be beneficial to improve childbirth outcomes.

## Key messages

- The small areas contribute the highest share of the total geographic variance of low birth weight (LBW) and small birth size (SBS) in India.
- A high burden of LBW is found mostly in the central‐western part of India and Odisha. The prevalence of SBS is high across the district of northern‐western regions and the north‐eastern regions of India.
- The mean prevalence and standard deviation are strongly correlated in the case of both LBW (*r* = 0.88) and SBS (*r* = 0.87) in India. It indicates that the districts which have a higher prevalence of LBW and SBS also have a higher between small area disparity within the districts.
- We find a similar pattern of distribution in LBW and SBS between the policy‐focused aspirational districts and other districts of India.
- Findings indicate reprioritizing the policy intervention, focusing on the small areas of India for better childbirth outcomes.

## Graphical abstract

The small areas contribute the highest share of the total geographic variance of low birth weight (LBW) and small birth size (SBS) in India. A high burden of LBW is found mostly in the central‐western part of India and Odisha, accompanied by a higher between small area disparity within the districts. Findings indicate reprioritizing the policy intervention, focusing on the small areas of India for better childbirth outcomes.

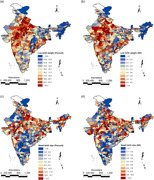

## 1 INTRODUCTION

Many studies estimated the district‐level prevalence of health indicators in India (International Institute for Population Sciences [IIPS] and ICF, 2017; Khan & Mohanty, 2018; Menon et al., 2018). The practice of estimating health indicators at the district level may mask inequalities between small areas, such as administrative blocks and villages within districts. Adopting a multilevel approach, recent studies have identified substantial small area variation within the district in a range of development and health indicators, such as body mass index (BMI) for women, poverty, child sex ratio and child nutrition (Kim et al., 2016, 2018, 2019; Mohanty et al., 2018; Rajpal et al., 2021; Rodgers et al., 2019). These findings support the supposition that there are small area inequalities within a relatively larger area like a district. For example, it was found that districts in India with a higher average prevalence of child undernutrition also have a higher level of inequalities within them (Rajpal et al., 2021).

Low birth weight (LBW) is an important indicator of the health of the mother and her children, and of the quality of maternal health care. It is a single measure that captures intrauterine growth influenced by several causes, from distal such as socioeconomic, environmental factors to proximal factors, such as maternal undernutrition, infections and clinical status (World Health Organisation [WHO], 2022). LBW increases the likelihood of several health complications, such as malnutrition, neurological disorders, respiratory suffering, hypoglycaemia and perinatal asphyxia (De Kieviet et al., 2009; Delobel‐Ayoub et al., 2006; Kramer, 1987; Marlow et al., 2005; Van Baar et al., 2005). It also increases the risk of lifelong illness and developmental disabilities, including diabetes, coronary heart diseases, high blood pressure, emotional distress and disabilities related to the physique, nervous system and intellect (Conde‐Agudelo et al., 2000; Wilson‐Costello et al., 2005).

Prior studies have identified a range of socioeconomic and demographic characteristics, such as caste groups, wealth status, use of maternity care and maternal nutritional status, in relation to increased risk of LBW in India (Bharati et al., 2011; Chakraborty & Anderson, 2011; Deshpande Jayant et al., 2011; Dharmalingam et al., 2010; International Institute for Population Sciences [IIPS] and ICF, 2017; Islam & Mohanty, 2021; Kader & Perera, 2014; Khan et al., 2020). Geographical factors can also help explain the within‐country differences in the prevalence of LBW (Yadav et al., 2015). Given the diversity of population characteristics and regional differences in India, it is highly likely that adverse birth outcomes like LBW will exhibit substantial geographic variation. While previous studies have mostly focused on the associations between socioeconomic and health factors with the risk of LBW, the analyses of geographical variance and small area variation have not been adequately considered in the previous studies (Balarajan et al., 2013; Banerjee et al., 2020; Bharati et al., 2011; Chakraborty & Anderson, 2011; Deshpande Jayant et al., 2011; Dharmalingam et al., 2010; Islam & Mohanty, 2021; Kader & Perera, 2014; Khan et al., 2020; Muthayya, 2009; Sen et al., 2010). Assessment of small area variation is needed to identify districts that have not only higher prevalence but also larger within‐district disparity. This identification of high burdened small areas within districts can help the policy makers and programme implementers to set priorities for effective policy intervention.

The Government of India launched the *Transformation of Aspirational Districts* programme that aims to efficiently transform districts that have a comparatively slower degree of progress in key development indicators (Ministry of Small Micro and Medium Enterprises, 2021; NITI Aayog, 2019). Accelerated efforts in the identified districts may help to significantly reduce inequalities across all districts of India, thereby putting the country on track towards achieving its stated sustainable development goals equitably and efficiently. Because LBW is a crucial indicator of health and development, investigating the prevalence of LBW and the inequality across small area units within these identified aspirational districts could meaningfully help in reshaping the policy formulation and implementation for better childbirth outcomes.

The reporting of birth weight has some limitations in developing countries like India. The birth weight for about one‐fifth of total births (22%) was not reported in the National Family Health Survey (NFHS) 2015–2016 (International Institute for Population Sciences [IIPS] and ICF, 2017). Since the proportion of reporting birth weight can vary considerably across states and by socioeconomic profile, the sample with complete data on birth weight may be biased. At the same time, the variable on reporting of birth size has negligible missing (including don't know) cases (<2%) (International Institute for Population Sciences [IIPS] and ICF, 2017). The small birth size (SBS) is the size of the baby at birth as reported by mothers as 'smaller than average' or 'very small'. Since babies reported to have big size at birth may also have higher birth weight, the estimates of birth size could be more meaningful in areas where the proportion of missing birth weight is relatively higher. So, there is a need to carry out a separate exercise for SBS in addition to the LBW.

The present study aims to estimate the multilevel geographical variance, precision‐weighted prevalence and small area variation of LBW and SBS in India. There are four specific objectives: (1) to partition the total geographic variation and estimate how much is attributable to the states, districts and small areas, (2) to assess the regional heterogeneity in the geographic variance explained by small areas across states and union territories, (3) to present the precision‐weighted prevalence and small area variation of LBW and SBS across the districts of India, and its correlation to variance across districts, and (4) to identify the policy‐prioritised districts in India based on the prevalence and small area variation.

## 2 DATA AND METHODS

### 2.1 Survey data and study population

**Figure 1:**
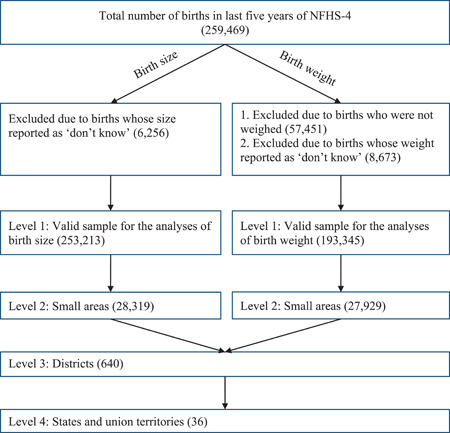
Schematic of the participant‐selection procedure with the hierarchy of sample distribution for birth weight and birth size in India, NFHS‐4 (2015–2016)

Our data has been drawn from the National Family Health Survey (NFHS‐4) conducted during 2015–2016 (International Institute for Population Sciences [IIPS] and ICF, 2017). This survey is equivalent to the globally known Demographic and Health Survey (DHS). It covers 640 districts nested within 29 states and 7 union territories. Of the 628,900 sampled households, 616,346 were occupied. Of the occupied households, the survey was conducted in 601,509 households with a response rate of 98%. Of the households who completed the survey, 723,875 eligible women aged 15–49 years were identified and 699,686 women completed the interviews with a response rate of 97%. Among the women interviewed, a total of 259,469 babies were born in the 5 years preceding the survey. After excluding the babies who were not weighed at the time of birth, as well as those whose birth weight information was not available at the time of the survey, 193,345 participants were included in the analysis of LBW. Similarly, a total of 253,213 births were included in the analyses of SBS after removing the cases whose birth sizes were ‘missing/don't know' (Figure 1).

### 2.2 Study design

This cross‐sectional survey used a two‐stage stratified random sampling framework. In the first stage, the primary sampling units (PSUs) were selected (International Institute for Population Sciences [IIPS] and ICF, 2017). The PSUs are villages in rural areas and census enumeration blocks (CEB) in urban areas. These PSUs are also known as clusters or communities (hereon we refer to them collectively as ‘small areas'). Within the selected small areas, households were chosen in the second stage. The data from the 2011 census was used as the sampling frame for selecting the small areas. The small areas with a small household size (<40) were merged with their nearest small areas. Six strata of small areas were prepared by intersecting three groups of small areas based on the number of households with two groups based on the proportion of scheduled caste/scheduled tribe population. Within each stratum, the small areas were sorted as per female literacy. The population proportion to size (PPS) sampling method was applied for selecting the small areas for sampling. If the number of households in the selected small areas was at least 300, the small areas were segmented into blocks with 100–150 households. Two of these segments were then selected for the sampling. In the second stage, a fixed number of 22 households were selected using systematic random sampling from each chosen small area.

### 2.3 Variables of interest

The main variables of interest in this study were LBW and SBS. The LBW was defined as children who weighed below 2.5 kg at the time of birth (World Health Organisation [WHO], 2006). Given the severity of the problem of LBW, the range was expanded to include very low birth weight (VLBW) and extremely low birth weight (ELBW) as secondary outcomes. The VLBW and ELBW were defined as the children born with less than 1.5 and 1.0 kg of birth weight, respectively (World Health Organisation [WHO], 2006). The birth weight was recorded in two ways during the survey. First, the birth weight was noted from the health card by the field investigators at the time of the survey. Second, it was recorded from the mothers' recall. For the variable of LBW, the records from both sources of information were combined.

For SBS, a question was posed to the mothers during the survey: *When (NAME) was born, was (he/she) very large, larger than average, average, smaller than average or very small?* From their responses, the variable of SBS was created. The SBS was defined as children who were reported as ‘very small' or ‘smaller than average' at the time of birth (coded as 1). The children who were reported as ‘average', ‘larger than average' or ‘large' were categorised as normal (coded as 0). To understand the severity of SBS, a secondary variable of very small birth size (VSBS) was also created and categorised into two groups–namely, VSBS, that is, ‘very small' (coded as 1) and normal birth size comprising the respondents of ‘smaller than average', ‘average', ‘larger than average' and ‘large' (coded as 0).

### 2.4 Statistical analyses

Four‐level logistic regression models were applied for partitioning the variance in LBW and SBS across the different levels. The levels of variance were children at level‐1 (*i*), small areas at level‐2 (*j*), districts at level‐3 (*k*) and states or union territories at level‐4 (*l*). Thus, the probability of LBW at these four levels can be predicted as: $Y_{\mathrm{ijkl}}=\beta_{0}+c_{0\mathrm{jkl}}+d_{0\mathrm{kl}}+s_{0l}$. In this model, $\beta_{0}$ is the constant and $c_{0\mathrm{jkl}}$, $d_{0\mathrm{kl}}$ and $s_{0l}$ are the residuals at the small area, district and state levels, respectively. The residuals are assumed to be normally distributed with mean 0 and variance of $\sigma_{c0}^{2}$, $\sigma_{d0}^{2}$ and $\sigma_{s0}^{2}$. These variances estimate between small areas within a district ($\sigma_{c0}^{2}$), between districts within a state ($\sigma_{d0}^{2}$) and between states within the country ($\sigma_{s0}^{2}$), respectively. A fixed individual‐level variance of $\pi^{2}/3$ or 3.29 can be assumed due to the logistic distribution of the outcome variable (Browne et al., 2005). The multilevel model was applied using the MLwiN 3.05 software programme via *runmlwin* command from STATA 16.0 (Browne et al., 2017; Leckie & Charlton, 2013). The Markov chain Monte Carlo (MCMC) was applied using the Gibbs sampling algorithm with default prior distributions of iterated generalised least square (IGLS) estimates as the starting values and monitoring of 5000 iterations after a burn‐in of 500 cycles.

The total geographic variance can be partitioned to level *z* as $\frac{\left(\sigma_{z0}^{2}\right)}{\left(\sigma_{c0}^{2}+\sigma_{d0}^{2}+\sigma_{s0}^{2}\right)}\times 100$. Here, the *z* is the different geographic level of variance, such as small areas ($\sigma_{c0}^{2}$), districts ($\sigma_{d0}^{2}$) and states ($\sigma_{s0}^{2}$). For the categorical variable, the variance partition for the individual level is fixed. We have done a separate exercise for variance partitioning of birth weight (continuous outcome) and found a similar variance partition attributable to the individual level considering 3.29 at the individual level in the case of the binary outcome (data not shown).

The state‐specific three‐level logistic regression models were applied to estimate the state‐wide geographic variances. The three levels of variance are children at level‐1 (*i*), small areas at level‐2 (*j*) and districts at level‐3 (*k*). Thus, the state‐specific probability of LBW and SBS at these three levels are estimated as $Y_{\mathrm{ijk}}=\beta_{0}+c_{0\mathrm{jk}}+d_{0k}$. Other assumptions of the model related to residual distribution and variance are the same as mentioned earlier. The state‐specific percentage of geographic variance attributable to the small areas has been estimated as $\left[\frac{\sigma_{c0}^{2}}{\left(\sigma_{c0}^{2}+\sigma_{d0}^{2}\right)}\right]\;*\;100$.

The precision‐weighted prevalence of LBW and SBS at the level of small areas were generated from the above‐described four‐level logistic regression models. In this process, the strengths of a higher sample size at the larger geographical levels (district and states) were borrowed for computing the precision‐weighted estimates to make them more reliable (Arcaya et al., 2012; Bell et al., 2019; Goldstein, 2011; Jones & Bullen, 1994; Leckie & Charlton, 2013; Subramanian et al., 2003). The resulting estimates are conservative and are inclined towards the mean values of districts and states. The precision‐weighted probability of LBW and SBS was multiplied by 100 to convert into the percentage. The probability of LBW and SBS for each small area was estimated as: $\frac{\exp\;\left(\beta_{0}+c_{0\mathrm{jkl}}+d_{0\mathrm{kl}}+s_{0l}\right)}{1+\exp\;\left(\beta_{0}+c_{0\mathrm{jkl}}+d_{0\mathrm{kl}}+s_{0l}\right)}$. The within‐district small area variation was computed as SDs of these prevalence estimates. The district‐wise probabilities were calculated as: $\frac{\exp\;\left(\beta_{0}+d_{0\mathrm{kl}}+s_{0l}\right)}{1+\exp\;(\beta_{0}+d_{0\mathrm{kl}}+s_{0l})}$.

The district‐level maps were prepared using ArcGIS Desktop 10.6. The shapefile for 640 districts was obtained from the Community Created Maps of India (CCMA) (http://projects.datameet.org/maps/).

## 3 RESULTS

### 3.1 Study participants and outcomes

In the selected study population, the prevalence of LBW is around 18% in India (Supporting Information: Table S1). The prevalence of VLBW and ELBW are <2% and <1%, respectively. About 12% and 3% of all children were reported as SBS and VSBS, respectively.

The information on LBW is derived from two sources (namely records from cards and mother's recall), while the information on SBS is the perception of mothers about the size of their children at birth. A sensitivity between LBW and SBS was 38%, with an ROC area of 61% showing a moderate correlation in reporting of SBS and LBW in India at the individual level. The district‐level correlation between LBW and SBS also showed a moderate association (*r* = 0.31) (Supporting Information: Figure S1).

### 3.2 Relative importance of multilevel geographies

**Figure 2:**
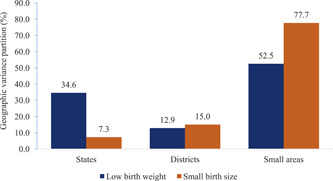
Geographic variance partitioning of low birth weight and small birth size in India, NFHS‐4 (2015–2016)

The variance partitioning from the four‐level logistic regression models shows that the largest share of geographical variance in both LBW and SBS are attributed to the small areas (Figure 2). Of the total geographical variance of LBW, the small area shares the largest variance of 53% (variance: 0.28; 95% confidence interval [CI]: 0.26–0.30), followed by the states with 35% (variance: 0.18; 95% CI: 0.11–0.31) and districts with 13% (variance: 0.07; 95% CI: 0.06–0.08) (Supporting Information: Table S2). Of the total variance in SBS, the total geographical variance at the level of small areas, districts and states are 78% (variance: 0.94; 95% CI: 0.89–0.99), 15% (variance: 0.18; 95% CI: 0.15–0.21) and 7% (variance: 0.09; 95% CI: 0.04–0.16), respectively.

**Figure 3:**
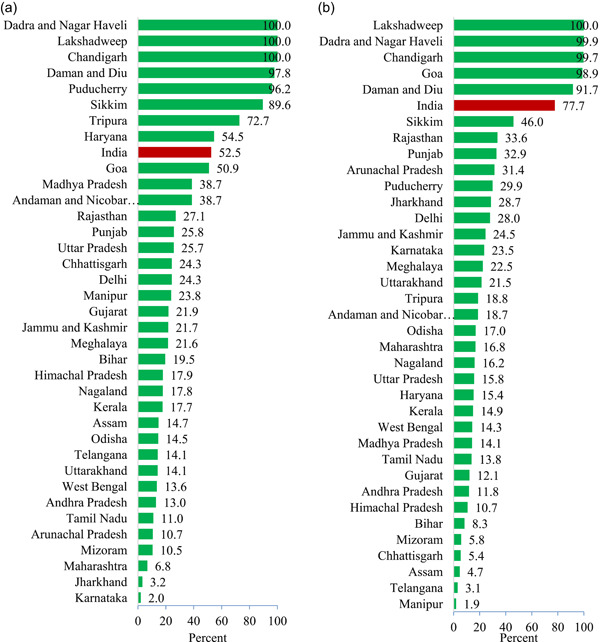
Geographic variance of (a) low birth weight and (b) small birth size attributable to the small areas (%) across the states and union territories of India, NFHS‐4 (2015–2016). Geographic variance for India includes the variance across the states, districts and small areas. Three union territories of India (Chandigarh, Lakshadweep and Dadra and Nagar Haveli) have only one district. Therefore, the geographic variance attributable to small areas is 100%

The geographic variance attributable to the small areas varies remarkably across the states and union territories of India in terms of both LBW and SBS (Figure 3). In the case of LBW, it ranges from 100% in Dadra and Nagar Haveli, Lakshadweep and Chandigarh to 2% in Karnataka (Figure 3A). In some of the small states and union territories, such as Daman and Diu, Puducherry, Sikkim and Tripura, the geographic variance attributable to the small areas is very high (73%–98%). Among the major states, Haryana (55%), Madhya Pradesh (39%), Rajasthan (27%), Punjab (26%) and Uttar Pradesh (26%) have relatively higher geographic variance attributable to the small areas as compared to states, such as Jharkhand (3%), Maharashtra (7%), Tamil Nadu (11%), West Bengal (14%), Uttarakhand (14%), Telangana (14%), Odisha (15%) and Assam (15%). In the majority of the remaining states and union territories, the variance attributable to the small areas lies between 15% and 25%.

### 3.3 Precision‐weighted estimates and small area variation

**Figure 4:**
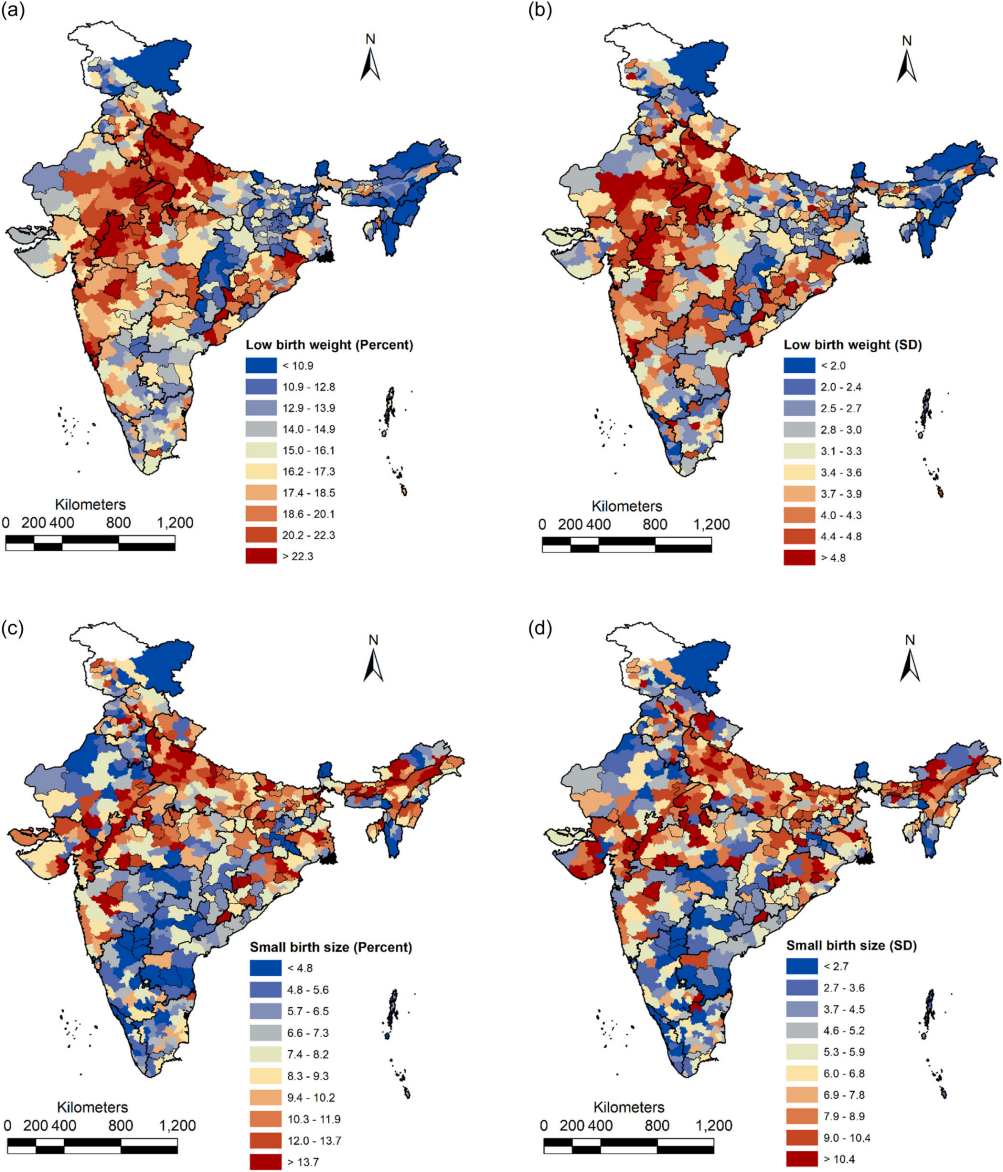
Geographic distribution of (a) prevalence of low birth weight (%), (b) within‐district, between‐small area standard deviation of low birth weight, (c) prevalence of small birth size (%) and (d) within‐district, between‐small area standard deviation of small birth size across 640 districts of India, NFHS‐4 (2015–2016)

The district‐level prevalence and within‐district, between‐small areas standard deviation (SD) were computed using the precision‐weighted estimates. The spatial distribution of the prevalence and small area variation (as measured by SD) across the districts are presented below (Figure 4), showing that there is a significant variation in the prevalence and SD of LBW across the districts and states of India. The prevalence of LBW ranges from <11% in the first decile to >22% in the last decile. A high burden of LBW (>16.2%) is found mostly in the central‐western part of India (Uttarakhand, Western Uttar Pradesh, part of Madhya Pradesh, Rajasthan, Gujarat and Maharashtra) and Odisha. The SD of LBW is also high in the districts of similar regions. Along similar lines, we found that the districts with a higher prevalence of SBS also had a higher within district SD.

### 3.4 Correlation between prevalence and small area variation

**Figure 5:**
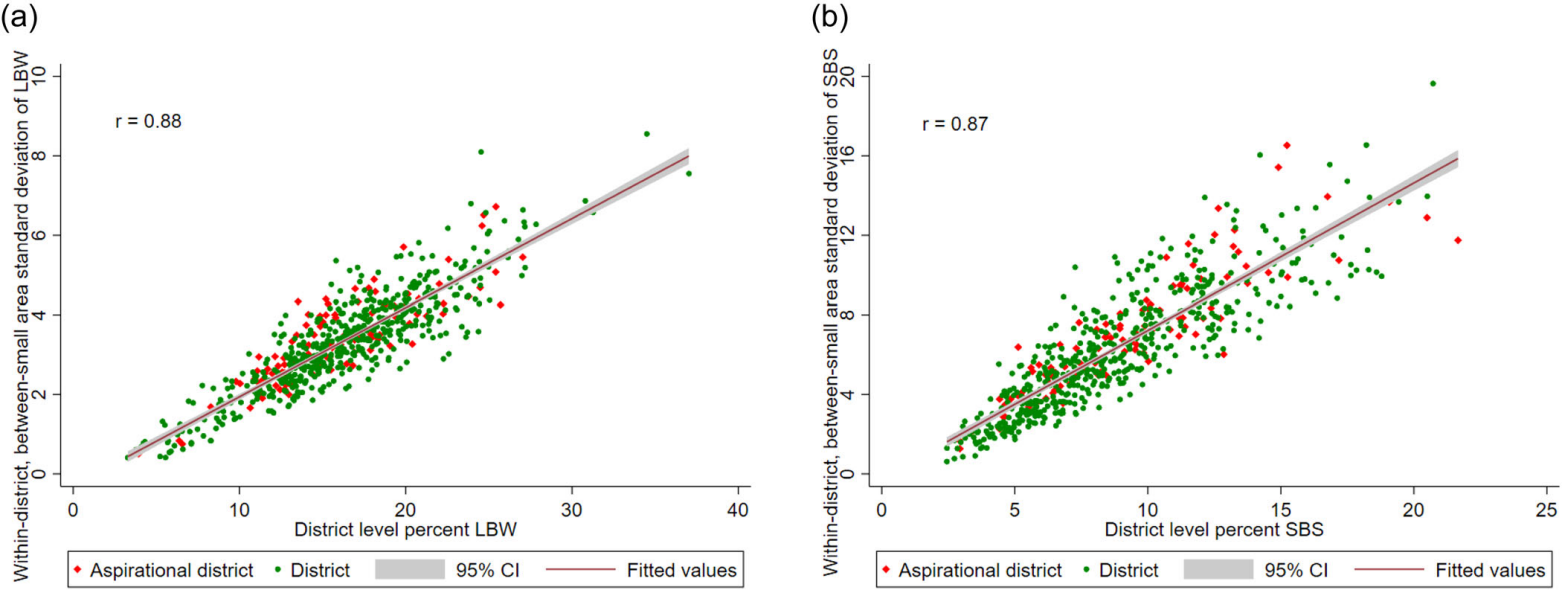
Correlation between the district level percent and within‐district, between‐small areas standard deviation of children of (a) low birth weight (LBW) and (b) small birth size (SBS) in India, NFHS‐4 (2015‐16)

The position of districts in relation to the district level prevalence and within‐district, between small area variation (SD), helps identify those districts that have a different level of prevalence with higher inequality in terms of LBW and SBS. Their relative positions reveal a strong association between prevalence and SD (*r* = 0.88) for LBW (Figure 5a). Importantly, the distribution of aspirational districts (in red) in terms of prevalence and SD is very similar to the nonaspirational districts (in green). Across the states and union territories of India, the correlation coefficients between prevalence and SD of the precision‐weighted percentage of LBW are significantly high with little variation, from the lowest in Maharashtra (*r* = 0.62) to the highest in Mizoram and Sikkim (*r* = 0.98) (Supporting Information: Table S3). The nonexistence of a negative correlation coefficient indicates that there are no states, which have low prevalence with high SD. Similarly, a strong correlation between prevalence and SD in SBS was found (Figure 5b).

### 3.5 Identification of policy focused districts

The total number of districts (640) was divided into three equal groups (tertile with 33.3% districts)—low, medium and high—based on the prevalence and SD, separately. Around 27% (174) of districts have a high prevalence with a high SD in LBW (Supporting Information: Table S4). Eight percent (51) of districts have a medium prevalence with a high SD. No district has a high prevalence with low SD. Out of the total number of districts, 27% (174) districts have a high prevalence with a high SD in SBS. The aspirational districts do not perform differently than the nonaspirational districts. A decile distribution of districts based on prevalence and SD is presented in Supporting Information: Table S5.

## 4 DISCUSSION

Of the total geographical variance among all administrative levels examined, we found that small areas have the highest share of variance in both LBW and SBS, differing greatly across India's states and union territories. Additionally, small area variation by districts shows large variation across the states and union territories. There is also a strong positive correlation between the prevalence and SD of LBW and SBS across all states and union territories. Finally, there is an insignificant difference in prevalence and SD between the policy‐focused aspirational districts and nonaspirational districts.

The small areas are the key units of consideration in this study since they have the highest level of geographical variance in India for adverse birth outcomes, such as LBW and SBS. Previous studies have also found that small areas play the largest role in total geographical variance for different health and development indicators (Kim et al., 2016, 2018, 2019; Mohanty et al., 2018; Rajpal et al., 2021; Rodgers et al., 2019). A higher geographic variance attributable to the small areas implies that small areas should be prioritised for achieving effective small area programme implementation, monitoring and governance. In the lower‐middle‐income countries like India, where the majority of areas have resource constraints, targeted strategies factoring small areas into the planning and intervention would accelerate progress towards achieving better birth outcomes. It is important to note that the geographic variance attributed to small areas also varied substantially across the states and union territories of India, indicating that this spatial heterogeneity needs to be considered when within‐district small areas are being prioritised. Currently, the decisions for resource allocation are made targeting the deprived districts. Within the district, small area variation in any diseases is not considered. Given the high concentration of LBW and SBS in select districts and its strong association with the small areas within districts, funding should be assigned contemplating the small area variation within the district.

There are substantial regional differentials in within‐district small area variation of LBW and SBS across the districts and states of India, which function as the main hindrance to equitable development. Well‐designed interventions in districts of high small area variation (inequality) would not only help lagging small areas improve but could also accelerate the overall progress of the district in the birth outcomes. Better understanding of these state‐wise differences in small area variation would help local governments more precisely prioritise their policy agenda. Since public health is a state concern as per the Seventh Schedule of the Constitution of India, state governments have a major role in framing new programmes and modifying existing ones.

The strong positive correlation between prevalence and SD of LBW and SBS across the districts of India demonstrates that districts with a higher level of prevalence also have higher within‐district, between‐small areas inequalities. A separate study finds similar patterns of distribution in child anthropometric failures (stunting, underweight and wasting) across the districts of India (Rajpal et al., 2021). Districts that have a low prevalence with high inequality would have critical policy challenges addressing within‐district small area variation. Although the number of districts showing low prevalence with high inequality is negligible, a significant number of districts with medium prevalence and high inequality were identified in the policy‐focused areas. Instances of high prevalence and high inequality are also key areas of policy concern.

The distribution of prevalence and SD of LBW and SBS across the aspirational districts is not different from that of the nonaspirational districts, implying that the problem of LBW and SBS prevails more uniformly across all districts than assumed in the programme. As India's aspirational district programme aims to accelerate key health indicators in underperforming districts, the current focus is on these target districts. However, our findings suggest the need for redefining the universe of aspirational districts. To that end, we have identified policy‐focused districts with different degrees of priority, factoring in the prevalence and small area variation for the indicators of adverse birth outcomes (Supporting Information: Table S5).

## 5 LIMITATIONS

This study had some limitations. Because about one‐fourth of the participants (25.4%) were excluded due to either nonmeasurement of weight at birth or reporting as ‘don't know', the unequal distribution of excluded participants across the states and union territories may affect the estimates (Supporting Information: Figure S2). Around half of the excluded samples were from Uttar Pradesh and Bihar. Thus, the estimates in these states may be disproportionately affected. Additionally, in the analyses of LBW, 403 small areas were excluded due to 100% of their cases being reported as missing. The education level and wealth status of respondents from these excluded small areas are significantly lower as compared to the respondents from the included small areas (Supporting Information: Table S6). Therefore, this exclusion of sample and small areas may slightly underestimate the prevalence of LBW in the larger administrative units (districts). Besides these excluded small areas, the sample size for some of the remaining small areas may also be reduced due to missing cases. In such small areas, the prevalence of LBW may be conservative and skewed towards the district and state mean prevalence. About half of the valid sample (47%) for birth weight was based upon mothers' recall and therefore subject to their recall bias towards digit preference in reporting. The digit preference emerges when mothers tend to report birth weight in certain digits (like 2000, 2500 and 3000 g). The geographical clustering of the birth weight reported may also have some impact on the estimated outcomes. Birth sizes may also have been subjected to the mothers' recall bias and self‐esteem rationalisation. These reasons may partly explain the weak to moderate consistency in reporting between LBW and SBS. Future research can look into the linkage of high LBW or SBS to caste and social class, anthropometric failures, prenatal care and other relevant factors for a synergistic and intersectoral response.

## 6 CONCLUSIONS

We put forward the critical need for precise policy attention to the small areas in formulating health programme interventions pertaining to reducing the adverse birth outcomes in India, as our findings demonstrate the existence of large within‐district, between‐small areas differentials in LBW and SBS. Mapping the estimates of the small area variations of LBW and SBS would help local governments improve and innovate on existing programmes in response to the need revealed by these discrepancies. Since the areas with high prevalence and high inequality of LBW and SBS are not necessarily concentrated in the aspirational districts, identifying districts with different degrees of prevalence with a high small area variation would help to reprioritise the most critical small area interventions in reducing the adverse childbirth outcomes. Efforts made in this direction may facilitate progress in reducing adverse birth outcomes in two ways. First, channelising programme inputs on the prenatal care services towards the most vulnerable small areas would diminish the small‐area variation within the districts and states by improving the birth outcomes in the small areas, which were lagging behind. Second, this catching‐up process of the lagged behind small areas to the advanced small areas would augment the overall progress towards achieving better birth outcomes in the states of India. As a result, it could significantly advance India's timeline for achieving the Sustainable Development Goals of health and well‐being for all.

## AUTHOR CONTRIBUTIONS

*Concept and design*: Rockli Kim and S. V.  Subramanian. *Acquisition, analysis or interpretation of data*: All authors. *Drafting of the manuscript*: Md Juel Rana. *Critical revision of the manuscript for important intellectual content*: All authors. *Statistical analysis*: Md Juel Rana. *Obtained funding*: Rockli Kim and S. V. Subramanian. *Administrative, technical or material support*: Rockli Kim and S. V. Subramanian. *Supervision*: Rockli Kim and S. V. Subramanian. Md Juel Rana had full access to all of the data in the study and takes responsibility for the integrity of the data and the accuracy of the data analysis.

## CONFLICTS OF INTEREST

The authors declare no conflicts of interest.

## ETHICS STATEMENT

This project used publicly accessible secondary data obtained from the DHS website. The DHS data are not collected specifically for this study and no one on the study team has access to identifiers linked to the data. These activities do not meet the regulatory definition of human subject research. As such, an Institutional Review Board (IRB) review is not required. The Harvard Longwood Campus IRB allows researchers to self‐determine when their research does not meet the requirements for IRB oversight using an IRB Decision Tool.

## Supporting information

Supporting information.

## Data Availability

DHS data are available at https://dhsprogram.com.
