# Supplementary material for: Small area variations in low birth weight and small size of births in India
Source: Matern Child Nutr. 2022 Apr 29;18(3):e13369. doi: 10.1111/mcn.13369 (PMC9218305; doi:10.1111/mcn.13369)
Supplement: Supplementary file 1 — Supporting information. [file MCN-18-e13369-s001.docx]

**Supplementary materials**

**Contents**

| **Table/Figure number** | **Title** | **Page number** |
| --- | --- | --- |
| Table S1 | Table S1: Descriptive statistics of the variables of interest in this study, NFHS-4 (2015-16) | 2 |
| Table S2 | Table S2: Variance of low birth weight and small birth size by different geographical levels in India | 3 |
| Table S3 | Table S3: The correlation coefficients between district level percent and within-district, between small areas standard deviation of children with low birth weight and small birth size across the states and union territories of India, NFHS-4 | 4 |
| Table S4 | Table S4: Distribution of districts based on the district level percent and within district, between small areas standard deviation of children with (a) low birth weight and (b) small birth size in India, NFHS-4 | 5 |
| Table S5 | Table S5: District level percent and within-district, between small area standard deviations (SD) of the children with low birth weight (LBW) and small birth size (SBS), and their positions in the decile distribution, India, 2015-16 | 6 |
| Table S6 | Table S6: Descriptive statistics of the selected and excluded PSUs for the analyses of birth weight | 32 |
| Figure S1 | Figure S1: Correlation between district level small birth size (%) and low birth weight (%) for 640 districts of India | 33 |
| Figure S2 | Figure S2: Sample distribution of the excluded participates in the analyses of birth weight | 34 |

Table S1: Descriptive statistics of the variables of interest in this study, NFHS-4 (2015-16)

| **Variables of interest** | **n** | **Percent** |
| --- | --- | --- |
| Birth weight |  |  |
| Normal (2.5 kg and above) | 159,228 | 82.4 |
| Low birth weight (<2.5 kg) | 34,117 | 17.6 |
| Birth weight |  |  |
| Normal (1.5 kg and above) | 190,827 | 98.7 |
| Very Low birth weight (<1.5 kg) | 2,518 | 1.3 |
| Birth weight |  |  |
| Normal (1.0 kg and above) | 193,099 | 99.9 |
| Extremely low birth weight (<1.0 kg) | 246 | 0.1 |
| **Total (birth weight)** | **193,345** | **100** |
| Birth size |  |  |
| Normal | 221,833 | 87.6 |
| Small birth size | 31,380 | 12.4 |
| Birth size |  |  |
| Normal | 245,234 | 96.8 |
| Very small birth size | 7,979 | 3.2 |
| **Total (birth size)** | **253,213** | **100** |

Note: n denotes sample size

Table S2: Variance of low birth weight and small birth size by different geographical levels in India

| Random effects | Low birth weight | Small birth size |
| --- | --- | --- |
| Constant | -1.72 [-1.83, -1.63] | -2.47 [-2.58, -2.36] |
| States | 0.18 [0.11, 0.31] | 0.09 [0.04, 0.16] |
| Districts | 0.07 [0.06, 0.08] | 0.18 [0.15, 0.21] |
| Small areas | 0.28 [0.26, 0.30] | 0.94 [0.89, 0.99] |

Note: A 95% confidence interval has been presented in the parentheses; the results have been derived from the four-level logit regression model since the variables have a binomial distribution

Table S3: The correlation coefficients between district level percent and within-district, between small areas standard deviation of children with low birth weight and small birth size across the states and union territories of India, NFHS-4

| States and Union Territories | Number of districts | Low birth weight | Small birth size |
| --- | --- | --- | --- |
| Andaman and Nicobar Islands | 3 | 0.83 | -0.86 |
| Andhra Pradesh | 13 | 0.93 | 0.90 |
| Arunachal Pradesh | 16 | 0.91 | 0.96 |
| Assam | 27 | 0.78 | 0.70 |
| Bihar | 38 | 0.79 | 0.85 |
| Chhattisgarh | 18 | 0.82 | 0.92 |
| Gujarat | 26 | 0.78 | 0.73 |
| Haryana | 21 | 0.81 | 0.89 |
| Himachal Pradesh | 12 | 0.83 | 0.90 |
| Jammu and Kashmir | 22 | 0.83 | 0.83 |
| Jharkhand | 24 | 0.75 | 0.91 |
| Karnataka | 30 | 0.68 | 0.88 |
| Kerala | 14 | 0.80 | 0.93 |
| Madhya Pradesh | 50 | 0.93 | 0.86 |
| Maharashtra | 35 | 0.62 | 0.84 |
| Manipur | 9 | 0.91 | 0.91 |
| Meghalaya | 7 | 0.84 | 0.98 |
| Mizoram | 8 | 0.98 | 0.85 |
| Nagaland | 11 | 0.82 | 0.90 |
| Delhi | 9 | 0.93 | 0.90 |
| Odisha | 30 | 0.81 | 0.87 |
| Puducherry | 4 | 0.92 | 0.84 |
| Punjab | 20 | 0.94 | 0.94 |
| Rajasthan | 33 | 0.83 | 0.89 |
| Sikkim | 4 | 0.98 | 0.85 |
| Tamil Nadu | 32 | 0.75 | 0.80 |
| Tripura | 4 | 0.78 | 0.76 |
| Uttar Pradesh | 71 | 0.82 | 0.76 |
| Uttarakhand | 13 | 0.73 | 0.85 |
| West Bengal | 19 | 0.77 | 0.89 |
| Telangana | 10 | 0.77 | 0.81 |
| Total | 640 | 0.88 | 0.87 |

Note: Lakshadweep, Chandigarh, and Dadra & Nagar Haveli were dropped due to only one observation. Daman & Diu and Goa have also been deleted because their correlation coefficient is 1, as these two administrative unit have only two observations (districts).

| Table S4: Distribution of districts based on the district level percent and within district, between small areas standard deviation of children with (a) low birth weight and (b) small birth size in India, NFHS-4  (a) Low birth weight | | | | |
| --- | --- | --- | --- | --- |
| All districts | Standard deviation | | | |
| Mean | Low | Medium | High | Total |
| Low | 169 | 41 | 4 | 214 |
| Medium | 42 | 120 | 51 | 213 |
| High | 0 | 39 | 174 | 213 |
| Total | 211 | 200 | 229 | 640 |
| Aspirational districts | Standard deviation | | | |
| Mean | Low | Medium | High | Total |
| Low | 33 | 9 | 2 | 44 |
| Medium | 5 | 22 | 12 | 39 |
| High | 0 | 5 | 24 | 29 |
| Total | 38 | 36 | 38 | 112 |
| Note: Low, medium and high are the tertile of mean or standard deviation. | | | | |

| (b) Small birth size | | | | |
| --- | --- | --- | --- | --- |
| All districts | Standard deviation | | | |
| Mean | Low | Medium | High | Total |
| Low | 169 | 44 | 1 | 214 |
| Medium | 45 | 130 | 38 | 213 |
| High | 0 | 39 | 174 | 213 |
| Total | 214 | 213 | 213 | 640 |
| Mean | Standard deviation | | | |
| Standard deviation | Low | Medium | High | Total |
| Low | 24 | 13 | 0 | 37 |
| Medium | 2 | 24 | 6 | 32 |
| High | 0 | 6 | 37 | 43 |
| Total | 26 | 43 | 43 | 112 |
| Note: Low, medium and high are the tertile of mean or standard deviation. | | | | |

Table S5: District level percent and within-district, between small area standard deviations (SD) of the children with low birth weight (LBW) and small birth size (SBS), and their positions in the decile distribution, India, 2015-16

| **Districts** | **States** | **Census 2011 District Code** | **Aspirational districts** | **Low birth weight (%)** | **Position of district in decile (LBW_%)** | **Low birth weight (SD)** | **Position of district in decile (LBW_SD)** | **Small birth size (%)** | **Position of district in decile (SBS_%)** | **Small birth size (SD)** | **Position of district in decile (SBS_SD)** |
| --- | --- | --- | --- | --- | --- | --- | --- | --- | --- | --- | --- |
| Data not available | Jammu & Kashmir | 0 | No | NA | NA | NA | NA | NA | NA | NA | NA |
| Kupwara | Jammu & Kashmir | 1 | Yes | 15.34 | 5th | 4.28 | 8th | 12.08 | 9th | 7.83 | 7th |
| Badgam | Jammu & Kashmir | 2 | No | 12.29 | 2nd | 2.56 | 3rd | 5.48 | 2nd | 4.5 | 4th |
| Leh | Jammu & Kashmir | 3 | No | 8.77 | 1st | 1.32 | 1st | 3.55 | 1st | 1.51 | 1st |
| Kargil | Jammu & Kashmir | 4 | No | 10.57 | 1st | 3.02 | 5th | 8.62 | 6th | 7.63 | 7th |
| Punch | Jammu & Kashmir | 5 | No | 16.63 | 6th | 5.12 | 10th | 10.04 | 7th | 8.03 | 8th |
| Rajouri | Jammu & Kashmir | 6 | No | 16.59 | 6th | 3.57 | 6th | 7.42 | 5th | 6.12 | 6th |
| Kathua | Jammu & Kashmir | 7 | No | 9.79 | 1st | 1.41 | 1st | 6.44 | 3rd | 3.9 | 3rd |
| Baramula | Jammu & Kashmir | 8 | Yes | 12.14 | 2nd | 2.96 | 4th | 9.55 | 7th | 7.08 | 7th |
| Bandipore | Jammu & Kashmir | 9 | No | 14.27 | 4th | 3.62 | 7th | 11.71 | 8th | 6.34 | 6th |
| Srinagar | Jammu & Kashmir | 10 | No | 12.97 | 3rd | 2.29 | 2nd | 5.77 | 3rd | 2.91 | 2nd |
| Ganderbal | Jammu & Kashmir | 11 | No | 13.03 | 3rd | 2.95 | 4th | 6.76 | 4th | 4.08 | 3rd |
| Pulwama | Jammu & Kashmir | 12 | No | 8.63 | 1st | 1.4 | 1st | 4.68 | 1st | 3.89 | 3rd |
| Shupiyan | Jammu & Kashmir | 13 | No | 12.03 | 2nd | 1.94 | 1st | 7.31 | 4th | 5.68 | 5th |
| Anantnag | Jammu & Kashmir | 14 | No | 14.51 | 4th | 2.87 | 4th | 10.42 | 8th | 5.47 | 5th |
| Kulgam | Jammu & Kashmir | 15 | No | 13.69 | 3rd | 2.46 | 3rd | 8.96 | 6th | 5.83 | 5th |
| Doda | Jammu & Kashmir | 16 | No | 13.37 | 3rd | 2.21 | 2nd | 8.3 | 6th | 4.96 | 4th |
| Ramban | Jammu & Kashmir | 17 | No | 18.08 | 7th | 3.62 | 7th | 3.45 | 1st | 2.17 | 1st |
| Kishtwar | Jammu & Kashmir | 18 | No | 15.97 | 5th | 3.82 | 7th | 4.6 | 1st | 2.34 | 1st |
| Udhampur | Jammu & Kashmir | 19 | No | 9.08 | 1st | 1.42 | 1st | 5.57 | 2nd | 3.99 | 3rd |
| Reasi | Jammu & Kashmir | 20 | No | 12.11 | 2nd | 3.51 | 6th | 12.14 | 9th | 13.9 | 10th |
| Jammu | Jammu & Kashmir | 21 | No | 10.92 | 2nd | 2.06 | 2nd | 7.79 | 5th | 4.05 | 3rd |
| Samba | Jammu & Kashmir | 22 | No | 13.16 | 3rd | 3.06 | 5th | 7.09 | 4th | 5.57 | 5th |
| Chamba | Himachal Pradesh | 23 | Yes | 14.95 | 4th | 2.78 | 4th | 9.58 | 7th | 6.51 | 6th |
| Kangra | Himachal Pradesh | 24 | No | 16.64 | 6th | 2.63 | 3rd | 7.26 | 4th | 3.96 | 3rd |
| Lahul And Spiti | Himachal Pradesh | 25 | No | 14.97 | 5th | 2.06 | 2nd | 7.57 | 5th | 3.31 | 2nd |
| Kullu | Himachal Pradesh | 26 | No | 15.39 | 5th | 2.25 | 2nd | 8.27 | 6th | 4.42 | 3rd |
| Mandi | Himachal Pradesh | 27 | No | 17.95 | 7th | 3.65 | 7th | 11.49 | 8th | 11 | 10th |
| Hamirpur | Himachal Pradesh | 28 | No | 21.44 | 9th | 3.86 | 7th | 15.08 | 10th | 11.37 | 10th |
| Una | Himachal Pradesh | 29 | No | 17.47 | 7th | 3.02 | 5th | 12.41 | 9th | 7.25 | 7th |
| Bilaspur | Himachal Pradesh | 30 | No | 18.82 | 8th | 4.36 | 9th | 8.97 | 6th | 4.56 | 4th |
| Solan | Himachal Pradesh | 31 | No | 19.18 | 8th | 3.81 | 7th | 15.01 | 10th | 10.02 | 9th |
| Sirmaur | Himachal Pradesh | 32 | No | 19.7 | 8th | 5.48 | 10th | 12.41 | 9th | 8.08 | 8th |
| Shimla | Himachal Pradesh | 33 | No | 17.19 | 6th | 2.67 | 3rd | 9.78 | 7th | 7.18 | 7th |
| Kinnaur | Himachal Pradesh | 34 | No | 12.99 | 3rd | 1.73 | 1st | 6.66 | 4th | 4.2 | 3rd |
| Gurdaspur | Punjab | 35 | No | 14.72 | 4th | 2.6 | 3rd | 6.46 | 3rd | 3.59 | 3rd |
| Kapurthala | Punjab | 36 | No | 12.71 | 2nd | 2.1 | 2nd | 4.96 | 2nd | 2.39 | 1st |
| Jalandhar | Punjab | 37 | No | 12.96 | 3rd | 2.57 | 3rd | 3.95 | 1st | 1.31 | 1st |
| Hoshiarpur | Punjab | 38 | No | 20.01 | 8th | 4.55 | 9th | 9.39 | 7th | 9.01 | 9th |
| Sangrur | Punjab | 39 | No | 16.2 | 6th | 2.68 | 3rd | 6.48 | 3rd | 3.68 | 3rd |
| Fatehgarh Sahib | Punjab | 40 | No | 20.8 | 9th | 4.08 | 8th | 5.99 | 3rd | 3.64 | 3rd |
| Ludhiana | Punjab | 41 | No | 17.16 | 6th | 3.71 | 7th | 13.72 | 10th | 9.73 | 9th |
| Moga | Punjab | 42 | Yes | 19.32 | 8th | 4.22 | 8th | 10.13 | 7th | 7.21 | 7th |
| Firozpur | Punjab | 43 | Yes | 17.08 | 6th | 3.45 | 6th | 9.84 | 7th | 6.08 | 6th |
| Muktsar | Punjab | 44 | No | 11.75 | 2nd | 2.21 | 2nd | 6.81 | 4th | 3.07 | 2nd |
| Faridkot | Punjab | 45 | No | 13.57 | 3rd | 2.72 | 3rd | 4.56 | 1st | 1.98 | 1st |
| Bathinda | Punjab | 46 | No | 14.54 | 4th | 2.94 | 4th | 8.38 | 6th | 6.54 | 6th |
| Mansa | Punjab | 47 | No | 20.57 | 9th | 4.69 | 9th | 12.97 | 9th | 8.12 | 8th |
| Patiala | Punjab | 48 | No | 20.51 | 9th | 4.83 | 10th | 12.42 | 9th | 8.58 | 8th |
| Amritsar | Punjab | 49 | No | 12.81 | 2nd | 2.07 | 2nd | 6.8 | 4th | 3.21 | 2nd |
| Tarn Taran | Punjab | 50 | No | 12.78 | 2nd | 3 | 5th | 5.15 | 2nd | 2.12 | 1st |
| Rupnagar | Punjab | 51 | No | 16.02 | 5th | 3.29 | 5th | 9.84 | 7th | 6.05 | 6th |
| Sahibzada Ajit Singh Nagar | Punjab | 52 | No | 17.5 | 7th | 4.08 | 8th | 9.87 | 7th | 7.06 | 7th |
| Shahid Bhagat Singh Nagar | Punjab | 53 | No | 19.08 | 8th | 4.3 | 9th | 12.67 | 9th | 7.31 | 7th |
| Barnala | Punjab | 54 | No | 19.71 | 8th | 4.23 | 8th | 7.85 | 5th | 4.9 | 4th |
| Chandigarh | Chandigarh | 55 | No | 20.02 | 8th | 4.32 | 9th | 8.52 | 6th | 4.7 | 4th |
| Uttarkashi | Uttarakhand | 56 | No | 23.71 | 10th | 3.56 | 6th | 10.87 | 8th | 11.33 | 10th |
| Chamoli | Uttarakhand | 57 | No | 19.65 | 8th | 2.65 | 3rd | 5.54 | 2nd | 2.67 | 2nd |
| Rudraprayag | Uttarakhand | 58 | No | 19.85 | 8th | 4.01 | 8th | 4.76 | 2nd | 2.15 | 1st |
| Tehri Garhwal | Uttarakhand | 59 | No | 23.37 | 10th | 3.7 | 7th | 11.77 | 8th | 11.27 | 10th |
| Dehradun | Uttarakhand | 60 | No | 18.85 | 8th | 3.81 | 7th | 7.66 | 5th | 4.77 | 4th |
| Garhwal | Uttarakhand | 61 | No | 20.06 | 8th | 3.34 | 6th | 12.13 | 9th | 10.28 | 9th |
| Pithoragarh | Uttarakhand | 62 | No | 21.38 | 9th | 3.77 | 7th | 9.8 | 7th | 4.5 | 4th |
| Bageshwar | Uttarakhand | 63 | No | 24.37 | 10th | 3.58 | 6th | 12.63 | 9th | 5.98 | 6th |
| Almora | Uttarakhand | 64 | No | 22.27 | 9th | 2.9 | 4th | 5.33 | 2nd | 3.12 | 2nd |
| Champawat | Uttarakhand | 65 | No | 26.19 | 10th | 5.6 | 10th | 12.12 | 9th | 9.41 | 9th |
| Nainital | Uttarakhand | 66 | No | 19.69 | 8th | 3.37 | 6th | 7.73 | 5th | 5.2 | 5th |
| Udham Singh Nagar | Uttarakhand | 67 | Yes | 27.04 | 10th | 5.45 | 10th | 13.75 | 10th | 9.58 | 9th |
| Hardwar | Uttarakhand | 68 | Yes | 25.41 | 10th | 5.07 | 10th | 12.98 | 9th | 9.9 | 9th |
| Panchkula | Haryana | 69 | No | 13.34 | 3rd | 2.31 | 2nd | 4.97 | 2nd | 2.33 | 1st |
| Ambala | Haryana | 70 | No | 13.11 | 3rd | 3.18 | 5th | 5.8 | 3rd | 3.14 | 2nd |
| Yamunanagar | Haryana | 71 | No | 17.98 | 7th | 3.21 | 5th | 7.66 | 5th | 7.79 | 7th |
| Kurukshetra | Haryana | 72 | No | 16.62 | 6th | 3.32 | 6th | 6.77 | 4th | 6.31 | 6th |
| Kaithal | Haryana | 73 | No | 17.51 | 7th | 3.38 | 6th | 5.58 | 2nd | 4.41 | 3rd |
| Karnal | Haryana | 74 | No | 14.64 | 4th | 3.28 | 5th | 4.61 | 1st | 4.17 | 3rd |
| Panipat | Haryana | 75 | No | 21.4 | 9th | 3.6 | 7th | 5.37 | 2nd | 4.24 | 3rd |
| Sonipat | Haryana | 76 | No | 24.5 | 10th | 4.92 | 10th | 3.14 | 1st | 1.81 | 1st |
| Jind | Haryana | 77 | No | 16.38 | 6th | 3.73 | 7th | 6.87 | 4th | 5.68 | 5th |
| Fatehabad | Haryana | 78 | No | 20.36 | 9th | 4.11 | 8th | 9.16 | 6th | 8.05 | 8th |
| Sirsa | Haryana | 79 | No | 18.45 | 7th | 4.31 | 9th | 8.29 | 6th | 4.52 | 4th |
| Hisar | Haryana | 80 | No | 14.48 | 4th | 3.27 | 5th | 6.16 | 3rd | 5.19 | 5th |
| Bhiwani | Haryana | 81 | No | 18.08 | 7th | 4.35 | 9th | 5.96 | 3rd | 4.26 | 3rd |
| Rohtak | Haryana | 82 | No | 17.08 | 6th | 3.62 | 7th | 9.96 | 7th | 9.24 | 9th |
| Jhajjar | Haryana | 83 | No | 16.73 | 6th | 3.89 | 7th | 7.27 | 4th | 10.39 | 9th |
| Mahendragarh | Haryana | 84 | No | 18.98 | 8th | 2.92 | 4th | 5.15 | 2nd | 2.98 | 2nd |
| Rewari | Haryana | 85 | No | 26.15 | 10th | 5.44 | 10th | 17.5 | 10th | 14.72 | 10th |
| Gurgaon | Haryana | 86 | No | 23.09 | 10th | 4.51 | 9th | 4.82 | 2nd | 4.21 | 3rd |
| Mewat | Haryana | 87 | Yes | 18.34 | 7th | 3.4 | 6th | 6.15 | 3rd | 4.99 | 4th |
| Faridabad | Haryana | 88 | No | 20.27 | 9th | 4.7 | 9th | 8.12 | 5th | 6.9 | 7th |
| Palwal | Haryana | 89 | No | 24.18 | 10th | 4.39 | 9th | 5.88 | 3rd | 6.44 | 6th |
| North West | Delhi | 90 | No | 27.84 | 10th | 6.28 | 10th | 6.87 | 4th | 5.6 | 5th |
| North | Delhi | 91 | No | 17.76 | 7th | 2.38 | 2nd | 11.03 | 8th | 5.96 | 6th |
| North East | Delhi | 92 | No | 26.99 | 10th | 4.98 | 10th | 9.93 | 7th | 6.8 | 6th |
| East | Delhi | 93 | No | 22.82 | 10th | 4.84 | 10th | 11.62 | 8th | 8.16 | 8th |
| New Delhi | Delhi | 94 | No | 20.03 | 8th | 3.71 | 7th | 6.55 | 4th | 2.43 | 1st |
| Central | Delhi | 95 | No | 20.59 | 9th | 3.74 | 7th | 5.96 | 3rd | 3.11 | 2nd |
| West | Delhi | 96 | No | 20.06 | 8th | 2.81 | 4th | 5.77 | 3rd | 2.55 | 1st |
| South West | Delhi | 97 | No | 21.63 | 9th | 3.43 | 6th | 5.72 | 3rd | 2.57 | 1st |
| South | Delhi | 98 | No | 18.18 | 7th | 2.92 | 4th | 6.66 | 4th | 2.64 | 2nd |
| Ganganagar | Rajasthan | 99 | No | 16.29 | 6th | 3.14 | 5th | 3.6 | 1st | 2.08 | 1st |
| Hanumangarh | Rajasthan | 100 | No | 14.53 | 4th | 2.45 | 3rd | 4.98 | 2nd | 2.61 | 2nd |
| Bikaner | Rajasthan | 101 | No | 13.26 | 3rd | 2.7 | 3rd | 3.59 | 1st | 2.82 | 2nd |
| Churu | Rajasthan | 102 | No | 15.52 | 5th | 2.84 | 4th | 7.96 | 5th | 6.03 | 6th |
| Jhunjhunun | Rajasthan | 103 | No | 15.3 | 5th | 3.36 | 6th | 4.88 | 2nd | 3.28 | 2nd |
| Alwar | Rajasthan | 104 | No | 20.1 | 9th | 4.2 | 8th | 3.52 | 1st | 1.61 | 1st |
| Bharatpur | Rajasthan | 105 | No | 20.79 | 9th | 5.81 | 10th | 5.57 | 2nd | 3.06 | 2nd |
| Dhaulpur | Rajasthan | 106 | Yes | 25.42 | 10th | 6.72 | 10th | 11.4 | 8th | 7.4 | 7th |
| Karauli | Rajasthan | 107 | Yes | 30.03 | 10th | 6.47 | 10th | 13.7 | 9th | 10.44 | 9th |
| Sawai Madhopur | Rajasthan | 108 | No | 27.05 | 10th | 6.64 | 10th | 10.31 | 8th | 9.76 | 9th |
| Dausa | Rajasthan | 109 | No | 24.92 | 10th | 6.04 | 10th | 13.04 | 9th | 8.37 | 8th |
| Jaipur | Rajasthan | 110 | No | 21.76 | 9th | 5.07 | 10th | 6.55 | 4th | 4.95 | 4th |
| Sikar | Rajasthan | 111 | No | 18.71 | 8th | 3.87 | 7th | 3.68 | 1st | 1.61 | 1st |
| Nagaur | Rajasthan | 112 | No | 21.41 | 9th | 5.07 | 10th | 7.46 | 5th | 6.58 | 6th |
| Jodhpur | Rajasthan | 113 | No | 21.3 | 9th | 4.99 | 10th | 5.38 | 2nd | 4.3 | 3rd |
| Jaisalmer | Rajasthan | 114 | Yes | 13.2 | 3rd | 2.78 | 4th | 6.2 | 3rd | 4.54 | 4th |
| Barmer | Rajasthan | 115 | No | 14.15 | 4th | 3.5 | 6th | 8.8 | 6th | 6.97 | 7th |
| Jalor | Rajasthan | 116 | No | 15.99 | 5th | 4.13 | 8th | 4.71 | 2nd | 4.04 | 3rd |
| Sirohi | Rajasthan | 117 | Yes | 15.48 | 5th | 3.27 | 5th | 8.96 | 6th | 8.07 | 8th |
| Pali | Rajasthan | 118 | No | 17.33 | 7th | 4.98 | 10th | 12.04 | 9th | 8.81 | 8th |
| Ajmer | Rajasthan | 119 | No | 18.74 | 8th | 4.31 | 9th | 3.3 | 1st | 2 | 1st |
| Tonk | Rajasthan | 120 | No | 23.08 | 10th | 4.13 | 8th | 14.13 | 10th | 9.03 | 9th |
| Bundi | Rajasthan | 121 | No | 18.55 | 8th | 3.84 | 7th | 12.83 | 9th | 6.29 | 6th |
| Bhilwara | Rajasthan | 122 | No | 18.7 | 8th | 3.4 | 6th | 7.37 | 5th | 4.46 | 3rd |
| Rajsamand | Rajasthan | 123 | No | 21.36 | 9th | 4.69 | 9th | 6.23 | 3rd | 3.41 | 2nd |
| Dungarpur | Rajasthan | 124 | No | 17.65 | 7th | 4.14 | 8th | 4.4 | 1st | 5.53 | 5th |
| Banswara | Rajasthan | 125 | No | 24.53 | 10th | 8.1 | 10th | 9.3 | 7th | 10.02 | 9th |
| Chittaurgarh | Rajasthan | 126 | No | 21.41 | 9th | 4.1 | 8th | 8.75 | 6th | 5.82 | 5th |
| Kota | Rajasthan | 127 | No | 18.32 | 7th | 3.29 | 5th | 13.82 | 10th | 9.95 | 9th |
| Baran | Rajasthan | 128 | Yes | 22.27 | 9th | 4.28 | 8th | 10.1 | 7th | 8.52 | 8th |
| Jhalawar | Rajasthan | 129 | No | 21.47 | 9th | 4.45 | 9th | 5.92 | 3rd | 4.77 | 4th |
| Udaipur | Rajasthan | 130 | No | 21.76 | 9th | 4.44 | 9th | 17.63 | 10th | 9.97 | 9th |
| Pratapgarh | Rajasthan | 131 | No | 24.49 | 10th | 4.74 | 9th | 6.4 | 3rd | 5.2 | 5th |
| Saharanpur | Uttar Pradesh | 132 | No | 22.66 | 10th | 4.71 | 9th | 18.79 | 10th | 9.93 | 9th |
| Muzaffarnagar | Uttar Pradesh | 133 | No | 24.81 | 10th | 6.57 | 10th | 15.79 | 10th | 9.75 | 9th |
| Bijnor | Uttar Pradesh | 134 | No | 24.18 | 10th | 5.18 | 10th | 9.32 | 7th | 6.29 | 6th |
| Moradabad | Uttar Pradesh | 135 | No | 27.18 | 10th | 5.18 | 10th | 18.6 | 10th | 10.14 | 9th |
| Rampur | Uttar Pradesh | 136 | No | 26.78 | 10th | 5.89 | 10th | 16.99 | 10th | 11 | 10th |
| Jyotiba Phule Nagar | Uttar Pradesh | 137 | No | 23.34 | 10th | 5.17 | 10th | 10.13 | 7th | 7.54 | 7th |
| Meerut | Uttar Pradesh | 138 | No | 22.95 | 10th | 4.5 | 9th | 17.12 | 10th | 8.83 | 8th |
| Baghpat | Uttar Pradesh | 139 | No | 20.61 | 9th | 3.86 | 7th | 8.73 | 6th | 5.07 | 4th |
| Ghaziabad | Uttar Pradesh | 140 | No | 21.34 | 9th | 3.95 | 8th | 13.26 | 9th | 7.57 | 7th |
| Gautam Buddha Nagar | Uttar Pradesh | 141 | No | 24.98 | 10th | 5.21 | 10th | 16.15 | 10th | 9.62 | 9th |
| Bulandshahr | Uttar Pradesh | 142 | No | 19.71 | 8th | 3.94 | 8th | 9.79 | 7th | 8.05 | 8th |
| Aligarh | Uttar Pradesh | 143 | No | 21.87 | 9th | 4.29 | 9th | 14.56 | 10th | 8.6 | 8th |
| Mahamaya Nagar | Uttar Pradesh | 144 | No | 24.1 | 10th | 5.68 | 10th | 17.63 | 10th | 10.52 | 10th |
| Mathura | Uttar Pradesh | 145 | No | 19.49 | 8th | 3.84 | 7th | 16.84 | 10th | 15.56 | 10th |
| Agra | Uttar Pradesh | 146 | No | 23.3 | 10th | 4.68 | 9th | 12.32 | 9th | 6.77 | 6th |
| Firozabad | Uttar Pradesh | 147 | No | 24.42 | 10th | 4.28 | 8th | 12.27 | 9th | 7.34 | 7th |
| Mainpuri | Uttar Pradesh | 148 | No | 20.51 | 9th | 4.15 | 8th | 20.72 | 10th | 19.64 | 10th |
| Budaun | Uttar Pradesh | 149 | No | 19.59 | 8th | 3.53 | 6th | 13.29 | 9th | 12.39 | 10th |
| Bareilly | Uttar Pradesh | 150 | No | 23.62 | 10th | 3.44 | 6th | 16.31 | 10th | 9.08 | 9th |
| Pilibhit | Uttar Pradesh | 151 | No | 19.07 | 8th | 3.62 | 7th | 14.18 | 10th | 6.84 | 7th |
| Shahjahanpur | Uttar Pradesh | 152 | No | 19.77 | 8th | 4.2 | 8th | 18.26 | 10th | 11.25 | 10th |
| Kheri | Uttar Pradesh | 153 | No | 24.12 | 10th | 4.86 | 10th | 9.88 | 7th | 8.33 | 8th |
| Sitapur | Uttar Pradesh | 154 | No | 26.21 | 10th | 5.54 | 10th | 12.26 | 9th | 9.53 | 9th |
| Hardoi | Uttar Pradesh | 155 | No | 22.53 | 10th | 6.18 | 10th | 10.6 | 8th | 6.92 | 7th |
| Unnao | Uttar Pradesh | 156 | No | 20.89 | 9th | 4.24 | 8th | 8.04 | 5th | 7.7 | 7th |
| Lucknow | Uttar Pradesh | 157 | No | 17.96 | 7th | 2.86 | 4th | 10.23 | 8th | 5.8 | 5th |
| Rae Bareli | Uttar Pradesh | 158 | No | 19.23 | 8th | 4.23 | 8th | 7.36 | 5th | 5.24 | 5th |
| Farrukhabad | Uttar Pradesh | 159 | No | 21.02 | 9th | 3.48 | 6th | 15.05 | 10th | 10.15 | 9th |
| Kannauj | Uttar Pradesh | 160 | No | 17.73 | 7th | 3.36 | 6th | 13.16 | 9th | 8.16 | 8th |
| Etawah | Uttar Pradesh | 161 | No | 20.8 | 9th | 4.8 | 9th | 12.52 | 9th | 7.94 | 8th |
| Auraiya | Uttar Pradesh | 162 | No | 18.26 | 7th | 2.76 | 4th | 10.39 | 8th | 6.37 | 6th |
| Kanpur Dehat | Uttar Pradesh | 163 | No | 22.43 | 10th | 3.54 | 6th | 7.94 | 5th | 6.5 | 6th |
| Kanpur Nagar | Uttar Pradesh | 164 | No | 16.17 | 6th | 2.53 | 3rd | 7.03 | 4th | 5.07 | 4th |
| Jalaun | Uttar Pradesh | 165 | No | 23.01 | 10th | 4.18 | 8th | 10.1 | 7th | 11.09 | 10th |
| Jhansi | Uttar Pradesh | 166 | No | 19.08 | 8th | 4.2 | 8th | 5.69 | 3rd | 3.11 | 2nd |
| Lalitpur | Uttar Pradesh | 167 | No | 19.52 | 8th | 4.5 | 9th | 10.55 | 8th | 11.83 | 10th |
| Hamirpur | Uttar Pradesh | 168 | No | 16.59 | 6th | 3.51 | 6th | 6.75 | 4th | 3.04 | 2nd |
| Mahoba | Uttar Pradesh | 169 | No | 18.79 | 8th | 4.13 | 8th | 7.38 | 5th | 5.65 | 5th |
| Banda | Uttar Pradesh | 170 | No | 15.09 | 5th | 2.73 | 3rd | 12.41 | 9th | 9.83 | 9th |
| Chitrakoot | Uttar Pradesh | 171 | Yes | 16.92 | 6th | 3.37 | 6th | 6.81 | 4th | 3.55 | 2nd |
| Fatehpur | Uttar Pradesh | 172 | Yes | 20.39 | 9th | 3.27 | 5th | 15.26 | 10th | 9.89 | 9th |
| Pratapgarh | Uttar Pradesh | 173 | No | 11.93 | 2nd | 1.58 | 1st | 7.05 | 4th | 4.44 | 3rd |
| Kaushambi | Uttar Pradesh | 174 | No | 14.93 | 4th | 2.96 | 4th | 7.85 | 5th | 7.28 | 7th |
| Allahabad | Uttar Pradesh | 175 | No | 14.63 | 4th | 2.76 | 4th | 10.47 | 8th | 10.9 | 10th |
| Bara Banki | Uttar Pradesh | 176 | No | 22.38 | 10th | 4.12 | 8th | 14.06 | 10th | 9.3 | 9th |
| Faizabad | Uttar Pradesh | 177 | No | 15.52 | 5th | 2.76 | 4th | 11.85 | 8th | 9.48 | 9th |
| Ambedkar Nagar | Uttar Pradesh | 178 | No | 16.15 | 6th | 3.72 | 7th | 11.29 | 8th | 8.21 | 8th |
| Sultanpur | Uttar Pradesh | 179 | No | 14.8 | 4th | 2.66 | 3rd | 7.59 | 5th | 7.11 | 7th |
| Bahraich | Uttar Pradesh | 180 | Yes | 25.7 | 10th | 4.25 | 8th | 19.07 | 10th | 13.66 | 10th |
| Shrawasti | Uttar Pradesh | 181 | Yes | 21.25 | 9th | 3.97 | 8th | 12.38 | 9th | 8.34 | 8th |
| Balrampur | Uttar Pradesh | 182 | Yes | 19.06 | 8th | 3.23 | 5th | 11.32 | 8th | 7.86 | 8th |
| Gonda | Uttar Pradesh | 183 | No | 23.02 | 10th | 4.82 | 10th | 12.57 | 9th | 8.66 | 8th |
| Siddharth Nagar | Uttar Pradesh | 184 | Yes | 19.33 | 8th | 3.98 | 8th | 10.02 | 7th | 5.66 | 5th |
| Basti | Uttar Pradesh | 185 | No | 16.75 | 6th | 3.46 | 6th | 11.7 | 8th | 8.13 | 8th |
| Sant Kabir Nagar | Uttar Pradesh | 186 | No | 15.5 | 5th | 2.98 | 4th | 10.06 | 7th | 8.04 | 8th |
| Mahrajganj | Uttar Pradesh | 187 | No | 18.16 | 7th | 3.7 | 7th | 9.09 | 6th | 5.62 | 5th |
| Gorakhpur | Uttar Pradesh | 188 | No | 14.74 | 4th | 2.9 | 4th | 11.85 | 8th | 8.58 | 8th |
| Kushinagar | Uttar Pradesh | 189 | No | 15.03 | 5th | 3.5 | 6th | 8.73 | 6th | 6.49 | 6th |
| Deoria | Uttar Pradesh | 190 | No | 13.65 | 3rd | 2.34 | 2nd | 8.75 | 6th | 5.73 | 5th |
| Azamgarh | Uttar Pradesh | 191 | No | 16.34 | 6th | 4.26 | 8th | 11.98 | 9th | 11.17 | 10th |
| Mau | Uttar Pradesh | 192 | No | 13.95 | 4th | 2.96 | 4th | 10.15 | 7th | 7.06 | 7th |
| Ballia | Uttar Pradesh | 193 | No | 14.84 | 4th | 3.9 | 7th | 8.29 | 6th | 7.87 | 8th |
| Jaunpur | Uttar Pradesh | 194 | No | 15.52 | 5th | 3.01 | 5th | 8.4 | 6th | 7.91 | 8th |
| Ghazipur | Uttar Pradesh | 195 | No | 13.6 | 3rd | 2.05 | 2nd | 10.15 | 7th | 7.09 | 7th |
| Chandauli | Uttar Pradesh | 196 | Yes | 16.45 | 6th | 2.77 | 4th | 10.01 | 7th | 8.32 | 8th |
| Varanasi | Uttar Pradesh | 197 | No | 17.88 | 7th | 3.89 | 7th | 11.92 | 9th | 9.4 | 9th |
| Sant Ravidas Nagar (Bhadohi) | Uttar Pradesh | 198 | No | 18.35 | 7th | 5.07 | 10th | 11.92 | 9th | 8.4 | 8th |
| Mirzapur | Uttar Pradesh | 199 | No | 14 | 4th | 2.34 | 2nd | 9.44 | 7th | 8.75 | 8th |
| Sonbhadra | Uttar Pradesh | 200 | Yes | 15.24 | 5th | 2.95 | 4th | 7.48 | 5th | 5.55 | 5th |
| Etah | Uttar Pradesh | 201 | No | 21.82 | 9th | 4.2 | 8th | 13.37 | 9th | 9.6 | 9th |
| Kanshiram Nagar | Uttar Pradesh | 202 | No | 22.02 | 9th | 3.84 | 7th | 18.31 | 10th | 10.27 | 9th |
| Pashchim Champaran | Bihar | 203 | No | 10.46 | 1st | 2.22 | 2nd | 11.87 | 8th | 9.87 | 9th |
| Purba Champaran | Bihar | 204 | No | 13.4 | 3rd | 2.34 | 2nd | 16.31 | 10th | 13.38 | 10th |
| Sheohar | Bihar | 205 | No | 16.3 | 6th | 3.47 | 6th | 10.14 | 7th | 7.88 | 8th |
| Sitamarhi | Bihar | 206 | Yes | 15.5 | 5th | 2.62 | 3rd | 7.98 | 5th | 5.95 | 6th |
| Madhubani | Bihar | 207 | No | 11.52 | 2nd | 2.44 | 3rd | 10.31 | 8th | 8.24 | 8th |
| Supaul | Bihar | 208 | No | 13.86 | 4th | 3.31 | 5th | 10.1 | 7th | 8.33 | 8th |
| Araria | Bihar | 209 | Yes | 12.78 | 2nd | 2.24 | 2nd | 9.95 | 7th | 8.73 | 8th |
| Kishanganj | Bihar | 210 | No | 9.45 | 1st | 1.95 | 2nd | 7.22 | 4th | 7.32 | 7th |
| Purnia | Bihar | 211 | Yes | 14.16 | 4th | 3.25 | 5th | 14.91 | 10th | 15.42 | 10th |
| Katihar | Bihar | 212 | Yes | 10.91 | 2nd | 2.39 | 3rd | 9.39 | 7th | 6.65 | 6th |
| Madhepura | Bihar | 213 | No | 10.81 | 1st | 2.33 | 2nd | 9.6 | 7th | 9.11 | 9th |
| Saharsa | Bihar | 214 | No | 11.43 | 2nd | 2.57 | 3rd | 7.93 | 5th | 5.89 | 5th |
| Darbhanga | Bihar | 215 | No | 16.76 | 6th | 4.31 | 9th | 11.28 | 8th | 10.01 | 9th |
| Muzaffarpur | Bihar | 216 | Yes | 16.82 | 6th | 2.73 | 3rd | 11.24 | 8th | 9.43 | 9th |
| Gopalganj | Bihar | 217 | No | 15.02 | 5th | 3.27 | 5th | 14.19 | 10th | 11.11 | 10th |
| Siwan | Bihar | 218 | No | 10.97 | 2nd | 2.34 | 2nd | 9.1 | 6th | 6.57 | 6th |
| Saran | Bihar | 219 | No | 14.3 | 4th | 3.54 | 6th | 10.71 | 8th | 7.68 | 7th |
| Vaishali | Bihar | 220 | No | 11.22 | 2nd | 2.12 | 2nd | 11.65 | 8th | 9.11 | 9th |
| Samastipur | Bihar | 221 | No | 13.33 | 3rd | 2.91 | 4th | 10.15 | 7th | 6.55 | 6th |
| Begusarai | Bihar | 222 | Yes | 15.02 | 5th | 3.83 | 7th | 11.17 | 8th | 6.94 | 7th |
| Khagaria | Bihar | 223 | Yes | 14.02 | 4th | 3.74 | 7th | 11.8 | 8th | 7.03 | 7th |
| Bhagalpur | Bihar | 224 | No | 9.57 | 1st | 2.19 | 2nd | 8.77 | 6th | 5.71 | 5th |
| Banka | Bihar | 225 | Yes | 9.8 | 1st | 2.33 | 2nd | 8.61 | 6th | 6.85 | 7th |
| Munger | Bihar | 226 | No | 11.68 | 2nd | 2.88 | 4th | 11.67 | 8th | 11.95 | 10th |
| Lakhisarai | Bihar | 227 | No | 15.45 | 5th | 4.16 | 8th | 15.85 | 10th | 11.86 | 10th |
| Sheikhpura | Bihar | 228 | Yes | 16.96 | 6th | 4.66 | 9th | 8.46 | 6th | 7.07 | 7th |
| Nalanda | Bihar | 229 | No | 17.24 | 6th | 4.83 | 10th | 11.88 | 8th | 8.61 | 8th |
| Patna | Bihar | 230 | No | 13.85 | 3rd | 3 | 5th | 6.24 | 3rd | 4.8 | 4th |
| Bhojpur | Bihar | 231 | No | 8.7 | 1st | 1.59 | 1st | 6.29 | 3rd | 5.08 | 4th |
| Buxar | Bihar | 232 | No | 10.81 | 1st | 2.56 | 3rd | 11.94 | 9th | 10.23 | 9th |
| Kaimur (Bhabua) | Bihar | 233 | No | 13.34 | 3rd | 3.41 | 6th | 11.88 | 8th | 8.68 | 8th |
| Rohtas | Bihar | 234 | No | 12.49 | 2nd | 3.78 | 7th | 9.58 | 7th | 6.82 | 6th |
| Aurangabad | Bihar | 235 | Yes | 16.36 | 6th | 3.51 | 6th | 14.53 | 10th | 10.12 | 9th |
| Gaya | Bihar | 236 | Yes | 14.86 | 4th | 3.71 | 7th | 11.28 | 8th | 9.56 | 9th |
| Nawada | Bihar | 237 | Yes | 12.68 | 2nd | 2.92 | 4th | 6.7 | 4th | 6.51 | 6th |
| Jamui | Bihar | 238 | Yes | 13.53 | 3rd | 4.33 | 9th | 6.88 | 4th | 5.4 | 5th |
| Jehanabad | Bihar | 239 | No | 14.44 | 4th | 3.21 | 5th | 8.26 | 6th | 7.18 | 7th |
| Arwal | Bihar | 240 | No | 11.02 | 2nd | 2.47 | 3rd | 6.35 | 3rd | 7.32 | 7th |
| North District | Sikkim | 241 | No | 6.09 | 1st | 0.71 | 1st | 3.05 | 1st | 0.86 | 1st |
| West District | Sikkim | 242 | Yes | 6.56 | 1st | 0.75 | 1st | 2.94 | 1st | 1.27 | 1st |
| South District | Sikkim | 243 | No | 7.48 | 1st | 1.13 | 1st | 2.84 | 1st | 1.28 | 1st |
| East District | Sikkim | 244 | No | 7.89 | 1st | 1.37 | 1st | 4.06 | 1st | 2 | 1st |
| Tawang | Arunachal Pradesh | 245 | No | 10.03 | 1st | 1.44 | 1st | 7.63 | 5th | 3.63 | 3rd |
| West Kameng | Arunachal Pradesh | 246 | No | 11.13 | 2nd | 2 | 2nd | 8.96 | 6th | 3.75 | 3rd |
| East Kameng | Arunachal Pradesh | 247 | No | 11.04 | 2nd | 2 | 2nd | 20.51 | 10th | 13.96 | 10th |
| Papumpare | Arunachal Pradesh | 248 | No | 10.92 | 2nd | 2.46 | 3rd | 11.37 | 8th | 7.23 | 7th |
| Upper Subansiri | Arunachal Pradesh | 249 | No | 7.11 | 1st | 0.79 | 1st | 5.57 | 2nd | 2.62 | 2nd |
| West Siang | Arunachal Pradesh | 250 | No | 8.27 | 1st | 0.84 | 1st | 4.51 | 1st | 3.31 | 2nd |
| East Siang | Arunachal Pradesh | 251 | No | 7.84 | 1st | 0.96 | 1st | 7.28 | 4th | 3.08 | 2nd |
| Upper Siang | Arunachal Pradesh | 252 | No | 8.29 | 1st | 0.84 | 1st | 6.1 | 3rd | 2.86 | 2nd |
| Changlang | Arunachal Pradesh | 253 | No | 9.62 | 1st | 1.53 | 1st | 7.37 | 5th | 4.85 | 4th |
| Tirap | Arunachal Pradesh | 254 | No | 8.97 | 1st | 1.25 | 1st | 8.17 | 5th | 4.93 | 4th |
| Lower Subansiri | Arunachal Pradesh | 255 | No | 8.98 | 1st | 1.6 | 1st | 9.33 | 7th | 4 | 3rd |
| Kurung Kumey | Arunachal Pradesh | 256 | No | 9.69 | 1st | 1.37 | 1st | 16.13 | 10th | 11.55 | 10th |
| Dibang Valley | Arunachal Pradesh | 257 | No | 11.06 | 2nd | 1.89 | 1st | 6.91 | 4th | 3.61 | 3rd |
| Lower Dibang Valley | Arunachal Pradesh | 258 | No | 8.6 | 1st | 1.15 | 1st | 15.64 | 10th | 10.8 | 10th |
| Lohit | Arunachal Pradesh | 259 | Yes | 11.3 | 2nd | 1.94 | 1st | 9.81 | 7th | 8.25 | 8th |
| Anjaw | Arunachal Pradesh | 260 | No | 11.8 | 2nd | 1.95 | 2nd | 10.37 | 8th | 7.32 | 7th |
| Mon | Nagaland | 261 | No | 7.77 | 1st | 2.23 | 2nd | 11.01 | 8th | 9.12 | 9th |
| Mokokchung | Nagaland | 262 | No | 5.23 | 1st | 0.44 | 1st | 7 | 4th | 4.04 | 3rd |
| Zunheboto | Nagaland | 263 | No | 6.6 | 1st | 0.62 | 1st | 8.14 | 5th | 3.99 | 3rd |
| Wokha | Nagaland | 264 | No | 5.78 | 1st | 0.54 | 1st | 8.61 | 6th | 5.66 | 5th |
| Dimapur | Nagaland | 265 | No | 8.77 | 1st | 1.75 | 1st | 10.66 | 8th | 7.72 | 7th |
| Phek | Nagaland | 266 | No | 5.56 | 1st | 0.41 | 1st | 4.53 | 1st | 3.95 | 3rd |
| Tuensang | Nagaland | 267 | No | 6.28 | 1st | 0.99 | 1st | 4.16 | 1st | 3.03 | 2nd |
| Longleng | Nagaland | 268 | No | 7.1 | 1st | 0.76 | 1st | 7.22 | 4th | 4.46 | 3rd |
| Kiphire | Nagaland | 269 | Yes | 6.38 | 1st | 0.84 | 1st | 7.69 | 5th | 5.25 | 5th |
| Kohima | Nagaland | 270 | No | 5.68 | 1st | 0.79 | 1st | 6.82 | 4th | 5.01 | 4th |
| Peren | Nagaland | 271 | No | 5.72 | 1st | 0.83 | 1st | 5.57 | 2nd | 3.67 | 3rd |
| Senapati (Excluding 3 Sub-Divisions) | Manipur | 272 | No | 6.43 | 1st | 1.25 | 1st | 12.09 | 9th | 7.87 | 8th |
| Tamenglong | Manipur | 273 | No | 6.55 | 1st | 1.07 | 1st | 12.03 | 9th | 9.12 | 9th |
| Churachandpur | Manipur | 274 | No | 7.02 | 1st | 1.07 | 1st | 9.17 | 6th | 4.83 | 4th |
| Bishnupur | Manipur | 275 | No | 7.21 | 1st | 1.79 | 1st | 9.25 | 6th | 5.27 | 5th |
| Thoubal | Manipur | 276 | No | 8.43 | 1st | 2.16 | 2nd | 10.71 | 8th | 6.32 | 6th |
| Imphal West | Manipur | 277 | No | 9.03 | 1st | 1.57 | 1st | 9.76 | 7th | 6.46 | 6th |
| Imphal East | Manipur | 278 | No | 8.86 | 1st | 1.87 | 1st | 11.24 | 8th | 7.66 | 7th |
| Ukhrul | Manipur | 279 | No | 5.88 | 1st | 0.57 | 1st | 10.25 | 8th | 6 | 6th |
| Chandel | Manipur | 280 | Yes | 11.17 | 2nd | 2.95 | 4th | 9.52 | 7th | 6.26 | 6th |
| Mamit | Mizoram | 281 | Yes | 3.93 | 1st | 0.51 | 1st | 4.58 | 1st | 2.87 | 2nd |
| Kolasib | Mizoram | 282 | No | 4.22 | 1st | 0.77 | 1st | 4.57 | 1st | 3.27 | 2nd |
| Aizawl | Mizoram | 283 | No | 5.52 | 1st | 1.23 | 1st | 7.16 | 4th | 4.65 | 4th |
| Champhai | Mizoram | 284 | No | 4.31 | 1st | 0.81 | 1st | 4.96 | 2nd | 4.31 | 3rd |
| Serchhip | Mizoram | 285 | No | 3.28 | 1st | 0.41 | 1st | 4.73 | 2nd | 3.62 | 3rd |
| Lunglei | Mizoram | 286 | No | 5.38 | 1st | 1.32 | 1st | 3.93 | 1st | 2.16 | 1st |
| Lawngtlai | Mizoram | 287 | No | 4.09 | 1st | 0.69 | 1st | 4.57 | 1st | 2.62 | 2nd |
| Saiha | Mizoram | 288 | No | 3.78 | 1st | 0.61 | 1st | 3.29 | 1st | 2.18 | 1st |
| West Tripura | Tripura | 289 | No | 16.01 | 5th | 2.96 | 4th | 10.13 | 7th | 6.82 | 6th |
| South Tripura | Tripura | 290 | No | 13.49 | 3rd | 1.95 | 2nd | 8.56 | 6th | 4.81 | 4th |
| Dhalai | Tripura | 291 | Yes | 12.79 | 2nd | 2.3 | 2nd | 10.84 | 8th | 7.26 | 7th |
| North Tripura | Tripura | 292 | No | 18.96 | 8th | 2.86 | 4th | 13.78 | 10th | 7.26 | 7th |
| West Garo Hills | Meghalaya | 293 | No | 13.86 | 4th | 3.19 | 5th | 5.85 | 3rd | 3.67 | 3rd |
| East Garo Hills | Meghalaya | 294 | No | 12.52 | 2nd | 3.46 | 6th | 13.23 | 9th | 12.77 | 10th |
| South Garo Hills | Meghalaya | 295 | No | 9.18 | 1st | 2.37 | 2nd | 5.7 | 3rd | 5.65 | 5th |
| West Khasi Hills | Meghalaya | 296 | No | 12.68 | 2nd | 3.51 | 6th | 4.96 | 2nd | 3.95 | 3rd |
| Ribhoi | Meghalaya | 297 | Yes | 11.23 | 2nd | 2.08 | 2nd | 4.86 | 2nd | 3.79 | 3rd |
| East Khasi Hills | Meghalaya | 298 | No | 10.19 | 1st | 2.53 | 3rd | 6.97 | 4th | 5.71 | 5th |
| Jaintia Hills | Meghalaya | 299 | No | 6.94 | 1st | 1.83 | 1st | 4.35 | 1st | 2.4 | 1st |
| Kokrajhar | Assam | 300 | No | 11.81 | 2nd | 2.15 | 2nd | 12.64 | 9th | 7.87 | 8th |
| Dhubri | Assam | 301 | Yes | 15.78 | 5th | 3.93 | 7th | 11.17 | 8th | 7.87 | 8th |
| Goalpara | Assam | 302 | Yes | 15.38 | 5th | 3.3 | 5th | 17.18 | 10th | 10.74 | 10th |
| Barpeta | Assam | 303 | Yes | 17.77 | 7th | 4.67 | 9th | 11.22 | 8th | 9.51 | 9th |
| Morigaon | Assam | 304 | No | 13.74 | 3rd | 3.47 | 6th | 15.43 | 10th | 10.6 | 10th |
| Nagaon | Assam | 305 | No | 13.08 | 3rd | 2.62 | 3rd | 11.78 | 8th | 10.74 | 10th |
| Sonitpur | Assam | 306 | No | 12.42 | 2nd | 1.85 | 1st | 14.33 | 10th | 12.45 | 10th |
| Lakhimpur | Assam | 307 | No | 13.14 | 3rd | 2.45 | 3rd | 12.11 | 9th | 8.64 | 8th |
| Dhemaji | Assam | 308 | No | 11.8 | 2nd | 2.36 | 2nd | 13.37 | 9th | 8.66 | 8th |
| Tinsukia | Assam | 309 | No | 17.53 | 7th | 3.96 | 8th | 15.61 | 10th | 13.35 | 10th |
| Dibrugarh | Assam | 310 | No | 17.31 | 7th | 3.38 | 6th | 14.67 | 10th | 9.71 | 9th |
| Sivasagar | Assam | 311 | No | 14.7 | 4th | 3.18 | 5th | 16.79 | 10th | 9.6 | 9th |
| Jorhat | Assam | 312 | No | 10.79 | 1st | 1.81 | 1st | 12.06 | 9th | 9.08 | 9th |
| Golaghat | Assam | 313 | No | 13.82 | 3rd | 2.93 | 4th | 13.12 | 9th | 10.11 | 9th |
| Karbi Anglong | Assam | 314 | No | 11.46 | 2nd | 2.03 | 2nd | 9.18 | 6th | 8.2 | 8th |
| Dima Hasao | Assam | 315 | No | 9.46 | 1st | 1.79 | 1st | 12.54 | 9th | 6.97 | 7th |
| Cachar | Assam | 316 | No | 14.72 | 4th | 3 | 5th | 11.74 | 8th | 8.59 | 8th |
| Karimganj | Assam | 317 | No | 12.78 | 2nd | 3.99 | 8th | 6.77 | 4th | 5.47 | 5th |
| Hailakandi | Assam | 318 | Yes | 11.42 | 2nd | 2.37 | 2nd | 6.41 | 3rd | 4.92 | 4th |
| Bongaigaon | Assam | 319 | No | 13.07 | 3rd | 2.88 | 4th | 14.85 | 10th | 8.44 | 8th |
| Chirang | Assam | 320 | No | 13.33 | 3rd | 2.5 | 3rd | 13.25 | 9th | 10.94 | 10th |
| Kamrup | Assam | 321 | No | 19.68 | 8th | 3.5 | 6th | 15.34 | 10th | 8.42 | 8th |
| Kamrup Metropolitan | Assam | 322 | No | 13.5 | 3rd | 2.07 | 2nd | 12.18 | 9th | 5.92 | 6th |
| Nalbari | Assam | 323 | No | 15.96 | 5th | 4.15 | 8th | 15.82 | 10th | 12.21 | 10th |
| Baksa | Assam | 324 | Yes | 14.14 | 4th | 3.02 | 5th | 9.06 | 6th | 6.75 | 6th |
| Darrang | Assam | 325 | Yes | 14.14 | 4th | 4.01 | 8th | 11.48 | 8th | 9.33 | 9th |
| Udalguri | Assam | 326 | Yes | 11.38 | 2nd | 1.91 | 1st | 10.95 | 8th | 9.45 | 9th |
| Darjiling | West Bengal | 327 | No | 14.35 | 4th | 2.17 | 2nd | 7.07 | 4th | 3.6 | 3rd |
| Jalpaiguri | West Bengal | 328 | No | 18.34 | 7th | 4.13 | 8th | 7.71 | 5th | 5.54 | 5th |
| Koch Bihar | West Bengal | 329 | No | 14.14 | 4th | 2.95 | 4th | 9.68 | 7th | 6.08 | 6th |
| Uttar Dinajpur | West Bengal | 330 | No | 14.7 | 4th | 3.89 | 7th | 15 | 10th | 8.92 | 8th |
| Dakshin Dinajpur | West Bengal | 331 | No | 13.33 | 3rd | 2.69 | 3rd | 7.71 | 5th | 5.02 | 4th |
| Maldah | West Bengal | 332 | No | 18.59 | 8th | 4.17 | 8th | 10.77 | 8th | 7.31 | 7th |
| Murshidabad | West Bengal | 333 | No | 14.3 | 4th | 2.92 | 4th | 7.52 | 5th | 5.15 | 4th |
| Birbhum | West Bengal | 334 | No | 13.69 | 3rd | 2.84 | 4th | 8.09 | 5th | 5.16 | 4th |
| Barddhaman | West Bengal | 335 | No | 19.83 | 8th | 3.81 | 7th | 14.82 | 10th | 11.78 | 10th |
| Nadia | West Bengal | 336 | No | 13.08 | 3rd | 2.75 | 4th | 10.47 | 8th | 7.99 | 8th |
| North Twenty Four Parganas | West Bengal | 337 | No | 14.31 | 4th | 2.52 | 3rd | 10.3 | 8th | 7.56 | 7th |
| Hugli | West Bengal | 338 | No | 19.27 | 8th | 3.14 | 5th | 12.1 | 9th | 6.4 | 6th |
| Bankura | West Bengal | 339 | No | 16.78 | 6th | 3.59 | 6th | 9.46 | 7th | 7.11 | 7th |
| Puruliya | West Bengal | 340 | No | 18.38 | 7th | 3.47 | 6th | 13.24 | 9th | 7.22 | 7th |
| Haora | West Bengal | 341 | No | 16.44 | 6th | 3.39 | 6th | 9.69 | 7th | 5.23 | 5th |
| Kolkata | West Bengal | 342 | No | 13.15 | 3rd | 2.37 | 2nd | 6.06 | 3rd | 2.57 | 1st |
| South Twenty Four Parganas | West Bengal | 343 | No | 13.37 | 3rd | 2.7 | 3rd | 5.77 | 3rd | 3.69 | 3rd |
| Paschim Medinipur | West Bengal | 344 | No | 17.11 | 6th | 3.59 | 6th | 8.26 | 6th | 5.47 | 5th |
| Purba Medinipur | West Bengal | 345 | No | 13.66 | 3rd | 2.44 | 3rd | 6.92 | 4th | 4.79 | 4th |
| Garhwa | Jharkhand | 346 | Yes | 12.19 | 2nd | 2.22 | 2nd | 8.43 | 6th | 4.98 | 4th |
| Chatra | Jharkhand | 347 | Yes | 13.17 | 3rd | 3.33 | 6th | 12.74 | 9th | 7.83 | 7th |
| Kodarma | Jharkhand | 348 | No | 12.4 | 2nd | 3.3 | 5th | 2.73 | 1st | 1.69 | 1st |
| Giridih | Jharkhand | 349 | Yes | 11.7 | 2nd | 2.64 | 3rd | 6.29 | 3rd | 4.6 | 4th |
| Deoghar | Jharkhand | 350 | No | 16.22 | 6th | 4.05 | 8th | 3.05 | 1st | 2.39 | 1st |
| Godda | Jharkhand | 351 | Yes | 12.57 | 2nd | 2.56 | 3rd | 5.67 | 3rd | 5.16 | 4th |
| Sahibganj | Jharkhand | 352 | Yes | 13.24 | 3rd | 2.19 | 2nd | 10.7 | 8th | 10.88 | 10th |
| Pakur | Jharkhand | 353 | Yes | 10.65 | 1st | 1.66 | 1st | 5.13 | 2nd | 6.38 | 6th |
| Dhanbad | Jharkhand | 354 | No | 15.08 | 5th | 3.16 | 5th | 8.73 | 6th | 7.38 | 7th |
| Bokaro | Jharkhand | 355 | Yes | 12.74 | 2nd | 2.12 | 2nd | 7.09 | 4th | 4.55 | 4th |
| Lohardaga | Jharkhand | 356 | Yes | 15.38 | 5th | 3.21 | 5th | 5.61 | 2nd | 5.35 | 5th |
| Purbi Singhbhum | Jharkhand | 357 | Yes | 12.44 | 2nd | 2.12 | 2nd | 4.64 | 1st | 3.49 | 2nd |
| Palamu | Jharkhand | 358 | Yes | 15.42 | 5th | 2.92 | 4th | 11.99 | 9th | 9.81 | 9th |
| Latehar | Jharkhand | 359 | Yes | 13.01 | 3rd | 2.27 | 2nd | 8.95 | 6th | 7.31 | 7th |
| Hazaribagh | Jharkhand | 360 | Yes | 12.33 | 2nd | 2.72 | 3rd | 5.14 | 2nd | 3.92 | 3rd |
| Ramgarh | Jharkhand | 361 | Yes | 12.86 | 3rd | 2.52 | 3rd | 11.7 | 8th | 10.5 | 10th |
| Dumka | Jharkhand | 362 | Yes | 15.75 | 5th | 4.01 | 8th | 7.42 | 5th | 7.62 | 7th |
| Jamtara | Jharkhand | 363 | No | 14.68 | 4th | 3.82 | 7th | 8.1 | 5th | 8.15 | 8th |
| Ranchi | Jharkhand | 364 | Yes | 14.36 | 4th | 2.52 | 3rd | 3.34 | 1st | 2.08 | 1st |
| Khunti | Jharkhand | 365 | Yes | 13.76 | 3rd | 3.25 | 5th | 5.34 | 2nd | 3.83 | 3rd |
| Gumla | Jharkhand | 366 | Yes | 14.48 | 4th | 3.5 | 6th | 12.51 | 9th | 12.03 | 10th |
| Simdega | Jharkhand | 367 | Yes | 12.97 | 3rd | 2 | 2nd | 5.53 | 2nd | 3.55 | 2nd |
| Pashchimi Singhbhum | Jharkhand | 368 | Yes | 11.97 | 2nd | 2.53 | 3rd | 8.14 | 5th | 4.98 | 4th |
| Saraikela Kharsawan | Jharkhand | 369 | No | 12.81 | 2nd | 2.32 | 2nd | 2.45 | 1st | 0.62 | 1st |
| Bargarh | Odisha | 370 | No | 16.93 | 6th | 3.67 | 7th | 5.89 | 3rd | 4.03 | 3rd |
| Jharsuguda | Odisha | 371 | No | 19.84 | 8th | 3.65 | 7th | 5.75 | 3rd | 3.27 | 2nd |
| Sambalpur | Odisha | 372 | No | 19.46 | 8th | 4.08 | 8th | 6.11 | 3rd | 3.21 | 2nd |
| Debagarh | Odisha | 373 | No | 15.12 | 5th | 3.01 | 5th | 11.21 | 8th | 9.23 | 9th |
| Sundargarh | Odisha | 374 | No | 17.03 | 6th | 3.41 | 6th | 12.13 | 9th | 9.74 | 9th |
| Kendujhar | Odisha | 375 | No | 21.64 | 9th | 4.6 | 9th | 15.56 | 10th | 10.61 | 10th |
| Mayurbhanj | Odisha | 376 | No | 25.05 | 10th | 4.37 | 9th | 8.67 | 6th | 6.17 | 6th |
| Baleshwar | Odisha | 377 | No | 20.35 | 9th | 3.73 | 7th | 6.57 | 4th | 5.07 | 4th |
| Bhadrak | Odisha | 378 | No | 20.43 | 9th | 4.79 | 9th | 13.8 | 10th | 8.03 | 8th |
| Kendrapara | Odisha | 379 | No | 17.73 | 7th | 3.83 | 7th | 7.82 | 5th | 6.58 | 6th |
| Jagatsinghapur | Odisha | 380 | No | 18.74 | 8th | 3.56 | 6th | 8.17 | 5th | 3.34 | 2nd |
| Cuttack | Odisha | 381 | No | 13.78 | 3rd | 2.71 | 3rd | 7.95 | 5th | 4.49 | 4th |
| Jajapur | Odisha | 382 | No | 19.87 | 8th | 4.03 | 8th | 11.21 | 8th | 8.16 | 8th |
| Dhenkanal | Odisha | 383 | Yes | 17.94 | 7th | 3.42 | 6th | 13.26 | 9th | 12.27 | 10th |
| Anugul | Odisha | 384 | No | 17.68 | 7th | 4.36 | 9th | 11.7 | 8th | 9.26 | 9th |
| Nayagarh | Odisha | 385 | No | 20.64 | 9th | 4.73 | 9th | 9.94 | 7th | 5.89 | 5th |
| Khordha | Odisha | 386 | No | 14.83 | 4th | 2.9 | 4th | 6.9 | 4th | 4.76 | 4th |
| Puri | Odisha | 387 | No | 20.31 | 9th | 5.53 | 10th | 8.56 | 6th | 6.6 | 6th |
| Ganjam | Odisha | 388 | No | 17.49 | 7th | 3.69 | 7th | 7.98 | 5th | 8.24 | 8th |
| Gajapati | Odisha | 389 | Yes | 14.56 | 4th | 2.91 | 4th | 6.46 | 3rd | 5.1 | 4th |
| Kandhamal | Odisha | 390 | Yes | 19.86 | 8th | 5.71 | 10th | 6.4 | 3rd | 5.17 | 5th |
| Baudh | Odisha | 391 | No | 16.68 | 6th | 2.86 | 4th | 13.33 | 9th | 13.23 | 10th |
| Subarnapur | Odisha | 392 | No | 17.65 | 7th | 4 | 8th | 11.91 | 9th | 8.08 | 8th |
| Balangir | Odisha | 393 | Yes | 22.02 | 9th | 4.78 | 9th | 20.5 | 10th | 12.89 | 10th |
| Nuapada | Odisha | 394 | Yes | 16.91 | 6th | 3.06 | 5th | 16.76 | 10th | 13.94 | 10th |
| Kalahandi | Odisha | 395 | Yes | 17.97 | 7th | 4.05 | 8th | 8.02 | 5th | 5.58 | 5th |
| Rayagada | Odisha | 396 | Yes | 20.69 | 9th | 4.02 | 8th | 12.85 | 9th | 6.02 | 6th |
| Nabarangapur | Odisha | 397 | Yes | 24.59 | 10th | 6.24 | 10th | 9.13 | 6th | 6.17 | 6th |
| Koraput | Odisha | 398 | Yes | 19.89 | 8th | 4.18 | 8th | 8.23 | 6th | 5.12 | 4th |
| Malkangiri | Odisha | 399 | Yes | 24.69 | 10th | 6.51 | 10th | 15.2 | 10th | 11.14 | 10th |
| Korea (Koriya) | Chhattisgarh | 400 | No | 11.6 | 2nd | 2.79 | 4th | 8.03 | 5th | 7.13 | 7th |
| Surguja | Chhattisgarh | 401 | No | 15.57 | 5th | 3.34 | 6th | 11.32 | 8th | 9.78 | 9th |
| Jashpur | Chhattisgarh | 402 | No | 15.77 | 5th | 3.58 | 6th | 8.11 | 5th | 6.37 | 6th |
| Raigarh | Chhattisgarh | 403 | No | 12.88 | 3rd | 2.93 | 4th | 6.28 | 3rd | 6.1 | 6th |
| Korba | Chhattisgarh | 404 | Yes | 11.18 | 2nd | 2.29 | 2nd | 10.45 | 8th | 8.24 | 8th |
| Janjgir - Champa | Chhattisgarh | 405 | No | 12.05 | 2nd | 1.54 | 1st | 5.79 | 3rd | 3.56 | 3rd |
| Bilaspur | Chhattisgarh | 406 | No | 9.55 | 1st | 2.33 | 2nd | 7.68 | 5th | 7.4 | 7th |
| Kabirdham | Chhattisgarh | 407 | No | 9.29 | 1st | 2.15 | 2nd | 7.64 | 5th | 5.79 | 5th |
| Rajnandgaon | Chhattisgarh | 408 | Yes | 10.04 | 1st | 2.28 | 2nd | 5.91 | 3rd | 5.5 | 5th |
| Durg | Chhattisgarh | 409 | No | 12.52 | 2nd | 2.33 | 2nd | 7.99 | 5th | 6.24 | 6th |
| Raipur | Chhattisgarh | 410 | No | 9.07 | 1st | 1.76 | 1st | 5.47 | 2nd | 3.54 | 2nd |
| Mahasamund | Chhattisgarh | 411 | Yes | 13.44 | 3rd | 3.49 | 6th | 7.31 | 4th | 6.33 | 6th |
| Dhamtari | Chhattisgarh | 412 | No | 12.39 | 2nd | 2.4 | 3rd | 5.5 | 2nd | 3 | 2nd |
| Uttar Bastar Kanker | Chhattisgarh | 413 | Yes | 15.2 | 5th | 4 | 8th | 5.52 | 2nd | 3.37 | 2nd |
| Bastar | Chhattisgarh | 414 | Yes | 11.52 | 2nd | 2.53 | 3rd | 5.83 | 3rd | 3.61 | 3rd |
| Narayanpur | Chhattisgarh | 415 | Yes | 11.07 | 2nd | 2.59 | 3rd | 8.43 | 6th | 7.54 | 7th |
| Dakshin Bastar Dantewada | Chhattisgarh | 416 | Yes | 14.45 | 4th | 2.89 | 4th | 6.72 | 4th | 4.42 | 3rd |
| Bijapur | Chhattisgarh | 417 | Yes | 8.28 | 1st | 1.68 | 1st | 5.27 | 2nd | 4.1 | 3rd |
| Sheopur | Madhya Pradesh | 418 | No | 27.12 | 10th | 6.33 | 10th | 12.43 | 9th | 11.7 | 10th |
| Morena | Madhya Pradesh | 419 | No | 21.93 | 9th | 4.28 | 8th | 6.83 | 4th | 8.9 | 8th |
| Bhind | Madhya Pradesh | 420 | No | 22.64 | 10th | 4.92 | 10th | 9.28 | 6th | 6.44 | 6th |
| Gwalior | Madhya Pradesh | 421 | No | 27.17 | 10th | 6.21 | 10th | 9.98 | 7th | 10.26 | 9th |
| Datia | Madhya Pradesh | 422 | No | 24.9 | 10th | 5.59 | 10th | 9.35 | 7th | 6.74 | 6th |
| Shivpuri | Madhya Pradesh | 423 | No | 21 | 9th | 5.42 | 10th | 9.62 | 7th | 10.25 | 9th |
| Tikamgarh | Madhya Pradesh | 424 | No | 24.43 | 10th | 5.33 | 10th | 12.45 | 9th | 7.51 | 7th |
| Chhatarpur | Madhya Pradesh | 425 | Yes | 18.74 | 8th | 4.25 | 8th | 7.32 | 5th | 4.99 | 4th |
| Panna | Madhya Pradesh | 426 | No | 21.16 | 9th | 4.89 | 10th | 9.54 | 7th | 7.3 | 7th |
| Sagar | Madhya Pradesh | 427 | No | 14.75 | 4th | 2.52 | 3rd | 10.87 | 8th | 6.45 | 6th |
| Damoh | Madhya Pradesh | 428 | Yes | 17.88 | 7th | 3.12 | 5th | 9.89 | 7th | 7.45 | 7th |
| Satna | Madhya Pradesh | 429 | No | 18.65 | 8th | 3.65 | 7th | 10.27 | 8th | 4.95 | 4th |
| Rewa | Madhya Pradesh | 430 | No | 22.09 | 9th | 3.48 | 6th | 6.55 | 4th | 4.42 | 3rd |
| Umaria | Madhya Pradesh | 431 | No | 14.74 | 4th | 2.81 | 4th | 7.55 | 5th | 4.33 | 3rd |
| Neemuch | Madhya Pradesh | 432 | No | 31.27 | 10th | 6.57 | 10th | 9.41 | 7th | 5.38 | 5th |
| Mandsaur | Madhya Pradesh | 433 | No | 37.03 | 10th | 7.55 | 10th | 19.43 | 10th | 13.68 | 10th |
| Ratlam | Madhya Pradesh | 434 | No | 30.8 | 10th | 6.86 | 10th | 17.28 | 10th | 11.91 | 10th |
| Ujjain | Madhya Pradesh | 435 | No | 25.94 | 10th | 6.36 | 10th | 10.44 | 8th | 8.18 | 8th |
| Shajapur | Madhya Pradesh | 436 | No | 21.17 | 9th | 4.52 | 9th | 9.63 | 7th | 6.95 | 7th |
| Dewas | Madhya Pradesh | 437 | No | 18.94 | 8th | 3.76 | 7th | 7.94 | 5th | 5.25 | 5th |
| Dhar | Madhya Pradesh | 438 | No | 23.57 | 10th | 5.34 | 10th | 8.65 | 6th | 6.56 | 6th |
| Indore | Madhya Pradesh | 439 | No | 20.39 | 9th | 4.17 | 8th | 6.71 | 4th | 5.88 | 5th |
| Khargone (West Nimar) | Madhya Pradesh | 440 | No | 19.5 | 8th | 4.19 | 8th | 18.34 | 10th | 13.9 | 10th |
| Barwani | Madhya Pradesh | 441 | Yes | 18.1 | 7th | 4.89 | 10th | 8.4 | 6th | 6.89 | 7th |
| Rajgarh | Madhya Pradesh | 442 | Yes | 20.23 | 9th | 3.79 | 7th | 8.43 | 6th | 6.89 | 7th |
| Vidisha | Madhya Pradesh | 443 | Yes | 23.78 | 10th | 4.46 | 9th | 13.4 | 9th | 11.16 | 10th |
| Bhopal | Madhya Pradesh | 444 | No | 19.58 | 8th | 3.6 | 7th | 8.01 | 5th | 5.89 | 5th |
| Sehore | Madhya Pradesh | 445 | No | 23.77 | 10th | 4.48 | 9th | 7.43 | 5th | 5.07 | 4th |
| Raisen | Madhya Pradesh | 446 | No | 15.05 | 5th | 2.8 | 4th | 12.97 | 9th | 13.55 | 10th |
| Betul | Madhya Pradesh | 447 | No | 18.41 | 7th | 3.94 | 8th | 8.42 | 6th | 7.12 | 7th |
| Harda | Madhya Pradesh | 448 | No | 21.6 | 9th | 5.48 | 10th | 7.54 | 5th | 8.06 | 8th |
| Hoshangabad | Madhya Pradesh | 449 | No | 17.55 | 7th | 4.12 | 8th | 6.12 | 3rd | 5.36 | 5th |
| Katni | Madhya Pradesh | 450 | No | 16.91 | 6th | 2.98 | 4th | 9.6 | 7th | 5.27 | 5th |
| Jabalpur | Madhya Pradesh | 451 | No | 16.24 | 6th | 3.09 | 5th | 5.56 | 2nd | 4.18 | 3rd |
| Narsimhapur | Madhya Pradesh | 452 | No | 21.54 | 9th | 3.94 | 8th | 14.24 | 10th | 7.69 | 7th |
| Dindori | Madhya Pradesh | 453 | No | 16.77 | 6th | 3.2 | 5th | 5.51 | 2nd | 3.54 | 2nd |
| Mandla | Madhya Pradesh | 454 | No | 16.82 | 6th | 3.02 | 5th | 5.85 | 3rd | 3 | 2nd |
| Chhindwara | Madhya Pradesh | 455 | No | 13.5 | 3rd | 2.84 | 4th | 4.24 | 1st | 1.81 | 1st |
| Seoni | Madhya Pradesh | 456 | No | 16.39 | 6th | 3.57 | 6th | 9.74 | 7th | 8.61 | 8th |
| Balaghat | Madhya Pradesh | 457 | No | 16.68 | 6th | 3.38 | 6th | 10.18 | 7th | 10.45 | 10th |
| Guna | Madhya Pradesh | 458 | Yes | 17.78 | 7th | 3.86 | 7th | 15.23 | 10th | 16.53 | 10th |
| Ashoknagar | Madhya Pradesh | 459 | No | 25.02 | 10th | 6.1 | 10th | 5.28 | 2nd | 3.53 | 2nd |
| Shahdol | Madhya Pradesh | 460 | No | 15.38 | 5th | 3.49 | 6th | 9.88 | 7th | 6.34 | 6th |
| Anuppur | Madhya Pradesh | 461 | No | 13.9 | 4th | 2.61 | 3rd | 6.52 | 4th | 3.06 | 2nd |
| Sidhi | Madhya Pradesh | 462 | No | 17.14 | 6th | 3.37 | 6th | 7.29 | 4th | 5.94 | 6th |
| Singrauli | Madhya Pradesh | 463 | Yes | 15.58 | 5th | 3.18 | 5th | 8.96 | 6th | 7.47 | 7th |
| Jhabua | Madhya Pradesh | 464 | No | 34.5 | 10th | 8.55 | 10th | 18.21 | 10th | 16.54 | 10th |
| Alirajpur | Madhya Pradesh | 465 | No | 23.92 | 10th | 6.8 | 10th | 14.22 | 10th | 16.04 | 10th |
| Khandwa (East Nimar) | Madhya Pradesh | 466 | Yes | 17.91 | 7th | 3.51 | 6th | 12.65 | 9th | 13.36 | 10th |
| Burhanpur | Madhya Pradesh | 467 | No | 20 | 8th | 4.07 | 8th | 5.95 | 3rd | 5.06 | 4th |
| Kachchh | Gujarat | 468 | No | 14.92 | 4th | 3.14 | 5th | 10.81 | 8th | 5.17 | 5th |
| Banaskantha | Gujarat | 469 | No | 17.9 | 7th | 4.57 | 9th | 12.02 | 9th | 9.41 | 9th |
| Patan | Gujarat | 470 | No | 17.64 | 7th | 3.4 | 6th | 12.31 | 9th | 9.65 | 9th |
| Mahesana | Gujarat | 471 | No | 17.3 | 6th | 3.12 | 5th | 9.97 | 7th | 6.79 | 6th |
| Sabarkantha | Gujarat | 472 | No | 17.16 | 6th | 3.67 | 7th | 11.24 | 8th | 7.47 | 7th |
| Gandhinagar | Gujarat | 473 | No | 17.16 | 6th | 3.2 | 5th | 7.24 | 4th | 4.79 | 4th |
| Ahmadabad | Gujarat | 474 | No | 16.59 | 6th | 2.21 | 2nd | 5 | 2nd | 1.74 | 1st |
| Surendranagar | Gujarat | 475 | No | 15.44 | 5th | 3.65 | 7th | 8.86 | 6th | 10.6 | 10th |
| Rajkot | Gujarat | 476 | No | 14.15 | 4th | 2.7 | 3rd | 8.69 | 6th | 8.84 | 8th |
| Jamnagar | Gujarat | 477 | No | 14.91 | 4th | 2.94 | 4th | 8.47 | 6th | 4.42 | 3rd |
| Porbandar | Gujarat | 478 | No | 14.44 | 4th | 2.7 | 3rd | 6.43 | 3rd | 3.57 | 3rd |
| Junagadh | Gujarat | 479 | No | 15.21 | 5th | 3.22 | 5th | 8.76 | 6th | 10.9 | 10th |
| Amreli | Gujarat | 480 | No | 16.24 | 6th | 3.07 | 5th | 10.87 | 8th | 9.37 | 9th |
| Bhavnagar | Gujarat | 481 | No | 18.49 | 8th | 3.84 | 7th | 15.03 | 10th | 13.02 | 10th |
| Anand | Gujarat | 482 | No | 18.23 | 7th | 3.49 | 6th | 9.17 | 6th | 5.22 | 5th |
| Kheda | Gujarat | 483 | No | 23.68 | 10th | 4.45 | 9th | 15.9 | 10th | 9.79 | 9th |
| Panchmahal | Gujarat | 484 | No | 20.49 | 9th | 4.81 | 9th | 14.43 | 10th | 12.24 | 10th |
| Dohad | Gujarat | 485 | Yes | 22.25 | 9th | 4.03 | 8th | 11.52 | 8th | 11.57 | 10th |
| Vadodara | Gujarat | 486 | No | 19.93 | 8th | 4.38 | 9th | 12.95 | 9th | 8.4 | 8th |
| Narmada | Gujarat | 487 | Yes | 24.48 | 10th | 4.68 | 9th | 21.66 | 10th | 11.75 | 10th |
| Bharuch | Gujarat | 488 | No | 18.82 | 8th | 4.63 | 9th | 9.9 | 7th | 6.84 | 7th |
| The Dangs | Gujarat | 489 | No | 19.05 | 8th | 3.49 | 6th | 14.74 | 10th | 10.56 | 10th |
| Navsari | Gujarat | 490 | No | 17.2 | 6th | 3.12 | 5th | 7.52 | 5th | 3.83 | 3rd |
| Valsad | Gujarat | 491 | No | 21.6 | 9th | 4.47 | 9th | 9.11 | 6th | 5.15 | 4th |
| Surat | Gujarat | 492 | No | 17.84 | 7th | 3.36 | 6th | 9.48 | 7th | 8.78 | 8th |
| Tapi | Gujarat | 493 | No | 16.89 | 6th | 3.06 | 5th | 12.41 | 9th | 10 | 9th |
| Diu | Daman and Diu | 494 | No | 15.42 | 5th | 2.67 | 3rd | 6.54 | 4th | 3.36 | 2nd |
| Daman | Daman and Diu | 495 | No | 17.3 | 6th | 3.54 | 6th | 4.54 | 1st | 2 | 1st |
| Dadra & Nagar Haveli | Dadra and Nagar Haveli | 496 | No | 20.58 | 9th | 4.69 | 9th | 5.09 | 2nd | 4.11 | 3rd |
| Nandurbar | Maharashtra | 497 | Yes | 18.43 | 7th | 3.5 | 6th | 6.17 | 3rd | 3.8 | 3rd |
| Dhule | Maharashtra | 498 | No | 18.19 | 7th | 3.26 | 5th | 8.91 | 6th | 9.72 | 9th |
| Jalgaon | Maharashtra | 499 | No | 19.44 | 8th | 5.46 | 10th | 6.18 | 3rd | 3.75 | 3rd |
| Buldana | Maharashtra | 500 | No | 20.07 | 9th | 4.13 | 8th | 6.93 | 4th | 3.98 | 3rd |
| Akola | Maharashtra | 501 | No | 15.61 | 5th | 3.83 | 7th | 5.44 | 2nd | 3.21 | 2nd |
| Washim | Maharashtra | 502 | Yes | 16.62 | 6th | 3.38 | 6th | 8.08 | 5th | 7.27 | 7th |
| Amravati | Maharashtra | 503 | No | 15.14 | 5th | 2.71 | 3rd | 3.84 | 1st | 1.74 | 1st |
| Wardha | Maharashtra | 504 | No | 14.8 | 4th | 2.86 | 4th | 4.01 | 1st | 2.01 | 1st |
| Nagpur | Maharashtra | 505 | No | 21.85 | 9th | 5.09 | 10th | 5.45 | 2nd | 2.97 | 2nd |
| Bhandara | Maharashtra | 506 | No | 21.02 | 9th | 3.74 | 7th | 5.21 | 2nd | 3.05 | 2nd |
| Gondiya | Maharashtra | 507 | No | 18.5 | 8th | 3.1 | 5th | 6.49 | 4th | 4.82 | 4th |
| Gadchiroli | Maharashtra | 508 | Yes | 20.13 | 9th | 4.52 | 9th | 6.52 | 4th | 5.11 | 4th |
| Chandrapur | Maharashtra | 509 | No | 18.26 | 7th | 3.3 | 5th | 4.64 | 1st | 2.54 | 1st |
| Yavatmal | Maharashtra | 510 | No | 18.92 | 8th | 3.92 | 7th | 6.54 | 4th | 7.48 | 7th |
| Nanded | Maharashtra | 511 | No | 14.49 | 4th | 2.97 | 4th | 4.77 | 2nd | 2.84 | 2nd |
| Hingoli | Maharashtra | 512 | No | 16.46 | 6th | 3.06 | 5th | 4.9 | 2nd | 5.06 | 4th |
| Parbhani | Maharashtra | 513 | No | 18.63 | 8th | 3.93 | 7th | 5.76 | 3rd | 5.9 | 6th |
| Jalna | Maharashtra | 514 | No | 18.83 | 8th | 4.7 | 9th | 6.41 | 3rd | 5.85 | 5th |
| Aurangabad | Maharashtra | 515 | Yes | 22.58 | 10th | 5.39 | 10th | 13.21 | 9th | 11.44 | 10th |
| Nashik | Maharashtra | 516 | No | 17.65 | 7th | 2.93 | 4th | 6.73 | 4th | 5.86 | 5th |
| Thane | Maharashtra | 517 | No | 20.44 | 9th | 4.14 | 8th | 7.74 | 5th | 4.23 | 3rd |
| Mumbai Suburban | Maharashtra | 518 | No | 15.67 | 5th | 2.34 | 2nd | 5.82 | 3rd | 3.37 | 2nd |
| Mumbai | Maharashtra | 519 | No | 19.27 | 8th | 4.4 | 9th | 7.2 | 4th | 5.81 | 5th |
| Raigarh | Maharashtra | 520 | No | 17.24 | 6th | 3.98 | 8th | 11.09 | 8th | 9.85 | 9th |
| Pune | Maharashtra | 521 | No | 17.53 | 7th | 3.9 | 7th | 7.82 | 5th | 6.32 | 6th |
| Ahmadnagar | Maharashtra | 522 | No | 21.75 | 9th | 4.49 | 9th | 17.84 | 10th | 10.24 | 9th |
| Bid | Maharashtra | 523 | No | 16.92 | 6th | 4 | 8th | 8.9 | 6th | 5.16 | 4th |
| Latur | Maharashtra | 524 | No | 14.49 | 4th | 2.84 | 4th | 6.22 | 3rd | 5.62 | 5th |
| Osmanabad | Maharashtra | 525 | Yes | 15.2 | 5th | 4.4 | 9th | 5.24 | 2nd | 3.59 | 3rd |
| Solapur | Maharashtra | 526 | No | 17.18 | 6th | 3.67 | 7th | 5.89 | 3rd | 2.93 | 2nd |
| Satara | Maharashtra | 527 | No | 17.38 | 7th | 3.1 | 5th | 8.39 | 6th | 5.81 | 5th |
| Ratnagiri | Maharashtra | 528 | No | 18.66 | 8th | 3.83 | 7th | 11.84 | 8th | 7.84 | 8th |
| Sindhudurg | Maharashtra | 529 | No | 22.59 | 10th | 3.02 | 5th | 8.02 | 5th | 5.66 | 5th |
| Kolhapur | Maharashtra | 530 | No | 20.85 | 9th | 4.99 | 10th | 9.75 | 7th | 5.31 | 5th |
| Sangli | Maharashtra | 531 | No | 17.48 | 7th | 3.74 | 7th | 12.77 | 9th | 9.6 | 9th |
| Adilabad | Telangana | 532 | Yes | 18.17 | 7th | 4.58 | 9th | 6.36 | 3rd | 5.35 | 5th |
| Nizamabad | Telangana | 533 | No | 17.95 | 7th | 4.21 | 8th | 5.15 | 2nd | 2.55 | 1st |
| Karimnagar | Telangana | 534 | No | 16.41 | 6th | 2.15 | 2nd | 8.03 | 5th | 5.12 | 4th |
| Medak | Telangana | 535 | No | 20.48 | 9th | 4.68 | 9th | 4.7 | 1st | 2.29 | 1st |
| Hyderabad | Telangana | 536 | No | 12.11 | 2nd | 2.62 | 3rd | 5.5 | 2nd | 5.04 | 4th |
| Rangareddy | Telangana | 537 | No | 16.47 | 6th | 3.6 | 7th | 6.2 | 3rd | 5.04 | 4th |
| Mahbubnagar | Telangana | 538 | No | 14.1 | 4th | 2.94 | 4th | 4.82 | 2nd | 3.32 | 2nd |
| Nalgonda | Telangana | 539 | No | 15.81 | 5th | 4.32 | 9th | 7.43 | 5th | 5.19 | 5th |
| Warangal | Telangana | 540 | Yes | 17.95 | 7th | 3.63 | 7th | 4.43 | 1st | 2.26 | 1st |
| Khammam | Telangana | 541 | Yes | 12.67 | 2nd | 2.11 | 2nd | 5.43 | 2nd | 3.65 | 3rd |
| Srikakulam | Andhra Pradesh | 542 | No | 13.01 | 3rd | 2.76 | 4th | 7.47 | 5th | 6.12 | 6th |
| Vizianagaram | Andhra Pradesh | 543 | Yes | 12.71 | 2nd | 2.54 | 3rd | 6.43 | 3rd | 4.07 | 3rd |
| Visakhapatnam | Andhra Pradesh | 544 | Yes | 16.86 | 6th | 3.57 | 6th | 6.48 | 3rd | 5.09 | 4th |
| East Godavari | Andhra Pradesh | 545 | No | 18.88 | 8th | 3.6 | 7th | 7.23 | 4th | 5.38 | 5th |
| West Godavari | Andhra Pradesh | 546 | No | 23.01 | 10th | 5.27 | 10th | 5.63 | 3rd | 4.06 | 3rd |
| Krishna | Andhra Pradesh | 547 | No | 14.35 | 4th | 2.84 | 4th | 6.92 | 4th | 4.72 | 4th |
| Guntur | Andhra Pradesh | 548 | No | 15.91 | 5th | 2.96 | 4th | 8 | 5th | 4.66 | 4th |
| Prakasam | Andhra Pradesh | 549 | No | 14.01 | 4th | 3.16 | 5th | 5.25 | 2nd | 3.91 | 3rd |
| Sri Potti Sriramulu Nellore | Andhra Pradesh | 550 | No | 17.05 | 6th | 4.34 | 9th | 4.11 | 1st | 2.33 | 1st |
| Y.S.R. | Andhra Pradesh | 551 | Yes | 14.31 | 4th | 2.91 | 4th | 4.43 | 1st | 3.76 | 3rd |
| Kurnool | Andhra Pradesh | 552 | No | 13.75 | 3rd | 2.66 | 3rd | 9.4 | 7th | 9.81 | 9th |
| Anantapur | Andhra Pradesh | 553 | No | 16.49 | 6th | 3.65 | 7th | 2.97 | 1st | 1.59 | 1st |
| Chittoor | Andhra Pradesh | 554 | No | 13.47 | 3rd | 2.72 | 3rd | 4.77 | 2nd | 2.34 | 1st |
| Belgaum | Karnataka | 555 | No | 17.52 | 7th | 4.62 | 9th | 7.12 | 4th | 5.63 | 5th |
| Bagalkot | Karnataka | 556 | No | 13.21 | 3rd | 2.73 | 3rd | 3.12 | 1st | 2.65 | 2nd |
| Bijapur | Karnataka | 557 | Yes | 14.85 | 4th | 3.97 | 8th | 4.62 | 1st | 2.89 | 2nd |
| Bidar | Karnataka | 558 | No | 16.07 | 5th | 4.6 | 9th | 6.22 | 3rd | 5.07 | 4th |
| Raichur | Karnataka | 559 | Yes | 12.54 | 2nd | 2.48 | 3rd | 3.47 | 1st | 2.35 | 1st |
| Koppal | Karnataka | 560 | No | 14.3 | 4th | 4 | 8th | 3.46 | 1st | 1.84 | 1st |
| Gadag | Karnataka | 561 | No | 13.59 | 3rd | 3.24 | 5th | 4 | 1st | 2.51 | 1st |
| Dharwad | Karnataka | 562 | No | 13.12 | 3rd | 2.86 | 4th | 7.24 | 4th | 8.38 | 8th |
| Uttara Kannada | Karnataka | 563 | No | 16.11 | 5th | 3.2 | 5th | 4.96 | 2nd | 3.45 | 2nd |
| Haveri | Karnataka | 564 | No | 16.12 | 6th | 4.03 | 8th | 3.73 | 1st | 3.04 | 2nd |
| Bellary | Karnataka | 565 | No | 17.84 | 7th | 4.53 | 9th | 6.48 | 3rd | 6.48 | 6th |
| Chitradurga | Karnataka | 566 | No | 13.68 | 3rd | 2.42 | 3rd | 4.44 | 1st | 2.28 | 1st |
| Davanagere | Karnataka | 567 | No | 15.04 | 5th | 2.73 | 3rd | 7.43 | 5th | 5.22 | 5th |
| Shimoga | Karnataka | 568 | No | 17.65 | 7th | 3.61 | 7th | 5.62 | 2nd | 3.15 | 2nd |
| Udupi | Karnataka | 569 | No | 20.17 | 9th | 4.4 | 9th | 9.09 | 6th | 6.03 | 6th |
| Chikmagalur | Karnataka | 570 | No | 17.73 | 7th | 3.4 | 6th | 6.69 | 4th | 3.64 | 3rd |
| Tumkur | Karnataka | 571 | No | 13.34 | 3rd | 2.67 | 3rd | 7.87 | 5th | 5.73 | 5th |
| Bangalore | Karnataka | 572 | No | 16.08 | 5th | 2.97 | 4th | 6.33 | 3rd | 6.38 | 6th |
| Mandya | Karnataka | 573 | No | 16.11 | 5th | 3.42 | 6th | 2.91 | 1st | 1.97 | 1st |
| Hassan | Karnataka | 574 | No | 15.33 | 5th | 2.59 | 3rd | 9.58 | 7th | 7.35 | 7th |
| Dakshina Kannada | Karnataka | 575 | No | 15 | 5th | 3.2 | 5th | 5.18 | 2nd | 5.43 | 5th |
| Kodagu | Karnataka | 576 | No | 18.52 | 8th | 3.9 | 7th | 2.73 | 1st | 0.77 | 1st |
| Mysore | Karnataka | 577 | No | 17.42 | 7th | 3.49 | 6th | 9.03 | 6th | 5.96 | 6th |
| Chamarajanagar | Karnataka | 578 | No | 13.78 | 3rd | 2.05 | 2nd | 8.29 | 6th | 5.1 | 4th |
| Gulbarga | Karnataka | 579 | No | 15.52 | 5th | 4.76 | 9th | 2.45 | 1st | 1.3 | 1st |
| Yadgir | Karnataka | 580 | Yes | 12.71 | 2nd | 2.75 | 4th | 2.9 | 1st | 1.71 | 1st |
| Kolar | Karnataka | 581 | No | 13.19 | 3rd | 2.18 | 2nd | 9.76 | 7th | 10.7 | 10th |
| Chikkaballapura | Karnataka | 582 | No | 15.24 | 5th | 3.27 | 5th | 5.36 | 2nd | 4.88 | 4th |
| Bangalore Rural | Karnataka | 583 | No | 12.73 | 2nd | 2.41 | 3rd | 8.97 | 6th | 7.94 | 8th |
| Ramanagara | Karnataka | 584 | No | 14.4 | 4th | 1.86 | 1st | 3.62 | 1st | 2.14 | 1st |
| North Goa | Goa | 585 | No | 21.29 | 9th | 4.57 | 9th | 5.52 | 2nd | 2.39 | 1st |
| South Goa | Goa | 586 | No | 23.51 | 10th | 5.22 | 10th | 9.31 | 7th | 6.51 | 6th |
| Lakshadweep | Lakshadweep | 587 | No | 17.11 | 6th | 5.19 | 10th | 11.44 | 8th | 9.78 | 9th |
| Kasaragod | Kerala | 588 | No | 13.35 | 3rd | 2.88 | 4th | 4.06 | 1st | 1.87 | 1st |
| Kannur | Kerala | 589 | No | 13.21 | 3rd | 1.89 | 1st | 3.51 | 1st | 0.91 | 1st |
| Wayanad | Kerala | 590 | Yes | 17.14 | 6th | 4.33 | 9th | 6.51 | 4th | 4.21 | 3rd |
| Kozhikode | Kerala | 591 | No | 13.33 | 3rd | 1.96 | 2nd | 4.16 | 1st | 2.02 | 1st |
| Malappuram | Kerala | 592 | No | 13.65 | 3rd | 2.41 | 3rd | 4.62 | 1st | 1.89 | 1st |
| Palakkad | Kerala | 593 | No | 18.09 | 7th | 4.1 | 8th | 7.8 | 5th | 6.24 | 6th |
| Thrissur | Kerala | 594 | No | 13.28 | 3rd | 1.85 | 1st | 4.99 | 2nd | 1.87 | 1st |
| Ernakulam | Kerala | 595 | No | 14.27 | 4th | 2.55 | 3rd | 5.17 | 2nd | 2.93 | 2nd |
| Idukki | Kerala | 596 | No | 12.94 | 3rd | 1.7 | 1st | 5.51 | 2nd | 2.39 | 1st |
| Kottayam | Kerala | 597 | No | 11.76 | 2nd | 1.59 | 1st | 3.99 | 1st | 2.03 | 1st |
| Alappuzha | Kerala | 598 | No | 14.88 | 4th | 2.11 | 2nd | 6.82 | 4th | 3.65 | 3rd |
| Pathanamthitta | Kerala | 599 | No | 17.98 | 7th | 2.62 | 3rd | 6.32 | 3rd | 4.46 | 3rd |
| Kollam | Kerala | 600 | No | 13.94 | 4th | 2.29 | 2nd | 5.81 | 3rd | 3.41 | 2nd |
| Thiruvananthapuram | Kerala | 601 | No | 15.91 | 5th | 3.4 | 6th | 6.53 | 4th | 3.51 | 2nd |
| Thiruvallur | Tamil Nadu | 602 | No | 14.78 | 4th | 2.54 | 3rd | 12.47 | 9th | 6.86 | 7th |
| Chennai | Tamil Nadu | 603 | No | 11.91 | 2nd | 1.99 | 2nd | 4.87 | 2nd | 2.18 | 1st |
| Kancheepuram | Tamil Nadu | 604 | No | 19.59 | 8th | 3.93 | 7th | 7.17 | 4th | 4.92 | 4th |
| Vellore | Tamil Nadu | 605 | No | 13.41 | 3rd | 2.21 | 2nd | 7.13 | 4th | 4.77 | 4th |
| Tiruvannamalai | Tamil Nadu | 606 | No | 14.69 | 4th | 2.62 | 3rd | 6.13 | 3rd | 2.81 | 2nd |
| Viluppuram | Tamil Nadu | 607 | No | 15.91 | 5th | 3.19 | 5th | 7.72 | 5th | 5.6 | 5th |
| Salem | Tamil Nadu | 608 | No | 13.55 | 3rd | 3.03 | 5th | 3.72 | 1st | 1.29 | 1st |
| Namakkal | Tamil Nadu | 609 | No | 15.81 | 5th | 5.36 | 10th | 5.25 | 2nd | 5.46 | 5th |
| Erode | Tamil Nadu | 610 | No | 14.42 | 4th | 2.66 | 3rd | 6.14 | 3rd | 6.41 | 6th |
| The Nilgiris | Tamil Nadu | 611 | No | 19.73 | 8th | 4.91 | 10th | 4.64 | 1st | 2.14 | 1st |
| Dindigul | Tamil Nadu | 612 | No | 14.32 | 4th | 2.67 | 3rd | 6.54 | 4th | 5.07 | 4th |
| Karur | Tamil Nadu | 613 | No | 12.91 | 3rd | 2.44 | 3rd | 5.12 | 2nd | 2.97 | 2nd |
| Tiruchirappalli | Tamil Nadu | 614 | No | 16.29 | 6th | 3.38 | 6th | 9.37 | 7th | 5.44 | 5th |
| Perambalur | Tamil Nadu | 615 | No | 18.27 | 7th | 3.71 | 7th | 7.3 | 4th | 3.01 | 2nd |
| Ariyalur | Tamil Nadu | 616 | No | 18.84 | 8th | 3.33 | 6th | 8.9 | 6th | 4.79 | 4th |
| Cuddalore | Tamil Nadu | 617 | No | 17.42 | 7th | 3.94 | 8th | 8.96 | 6th | 7.85 | 8th |
| Nagapattinam | Tamil Nadu | 618 | No | 17.37 | 7th | 3.15 | 5th | 9.08 | 6th | 4.59 | 4th |
| Thiruvarur | Tamil Nadu | 619 | No | 17.41 | 7th | 3.68 | 7th | 8.21 | 6th | 5.67 | 5th |
| Thanjavur | Tamil Nadu | 620 | No | 13.9 | 4th | 2.71 | 3rd | 9.9 | 7th | 7.47 | 7th |
| Pudukkottai | Tamil Nadu | 621 | No | 16.11 | 5th | 3.07 | 5th | 6.67 | 4th | 3.1 | 2nd |
| Sivaganga | Tamil Nadu | 622 | No | 12.56 | 2nd | 2.29 | 2nd | 5.89 | 3rd | 4.91 | 4th |
| Madurai | Tamil Nadu | 623 | No | 16.91 | 6th | 3.05 | 5th | 9.93 | 7th | 7.8 | 7th |
| Theni | Tamil Nadu | 624 | No | 16.04 | 5th | 2.99 | 5th | 4.88 | 2nd | 2.09 | 1st |
| Virudhunagar | Tamil Nadu | 625 | Yes | 20.79 | 9th | 4.41 | 9th | 6.44 | 3rd | 4.97 | 4th |
| Ramanathapuram | Tamil Nadu | 626 | Yes | 16.09 | 5th | 3.26 | 5th | 8.15 | 5th | 6.32 | 6th |
| Thoothukkudi | Tamil Nadu | 627 | No | 16.08 | 5th | 2.8 | 4th | 8.32 | 6th | 5.67 | 5th |
| Tirunelveli | Tamil Nadu | 628 | No | 15.44 | 5th | 2.88 | 4th | 5.28 | 2nd | 2.69 | 2nd |
| Kanniyakumari | Tamil Nadu | 629 | No | 15.88 | 5th | 3.07 | 5th | 6.15 | 3rd | 4.63 | 4th |
| Dharmapuri | Tamil Nadu | 630 | No | 12.02 | 2nd | 2.08 | 2nd | 3.79 | 1st | 1.35 | 1st |
| Krishnagiri | Tamil Nadu | 631 | No | 16.83 | 6th | 4.45 | 9th | 10.03 | 7th | 10.47 | 10th |
| Coimbatore | Tamil Nadu | 632 | No | 15.17 | 5th | 3.73 | 7th | 8.37 | 6th | 5.27 | 5th |
| Tiruppur | Tamil Nadu | 633 | No | 13.5 | 3rd | 2.79 | 4th | 4.44 | 1st | 2.54 | 1st |
| Yanam | Puducherry | 634 | No | 19.96 | 8th | 4.52 | 9th | 7.25 | 4th | 3.53 | 2nd |
| Puducherry | Puducherry | 635 | No | 15.07 | 5th | 2.19 | 2nd | 5.16 | 2nd | 2.65 | 2nd |
| Mahe | Puducherry | 636 | No | 10.09 | 1st | 1.8 | 1st | 7.74 | 5th | 5.25 | 5th |
| Karaikal | Puducherry | 637 | No | 16.13 | 6th | 3.41 | 6th | 5.26 | 2nd | 3.25 | 2nd |
| Nicobars | Andaman and Nicobar Islands | 638 | No | 17.93 | 7th | 4.4 | 9th | 5.63 | 3rd | 3.52 | 2nd |
| North & Middle Andaman | Andaman and Nicobar Islands | 639 | No | 15.92 | 5th | 2.68 | 3rd | 5.94 | 3rd | 3.07 | 2nd |
| South Andaman | Andaman and Nicobar Islands | 640 | No | 13.86 | 4th | 2.81 | 4th | 4.09 | 1st | 3.77 | 3rd |

Note: The position of the districts in the decile distribution were placed in the right column of each indicator.

| Table S6: Descriptive statistics of the selected and excluded PSUs for the analyses of birth weight | | | | | |
| --- | --- | --- | --- | --- | --- |
| Indicators | Number of PSUs | Mean | SD | Minimum | Maximum |
| All PSUs |  |  |  |  |  |
| Mean years of schooling | 28,332 | 7.1 | 3.7 | 0.0 | 20.0 |
| Mean wealth score | 28,332 | 2322 | 82888 | -182963 | 256176 |
| Selected PSUs |  |  |  |  |  |
| Mean years of schooling | 27,929 | 7.1 | 3.6 | 0.0 | 20.0 |
| Mean wealth score | 27,929 | 3284 | 82722 | -182963 | 256176 |
| Excluded PSUs |  |  |  |  |  |
| Mean years of schooling | 403 | 4.0 | 3.4 | 0.0 | 15.0 |
| Mean wealth score | 403 | -64332 | 65399 | -181018.8 | 201191 |
| Note: Mean wealth score is the calculated score from a range of household assets (IIPS & ICF, 2017) | | | | | |

Figure S1: Correlation between district level small birth size (%) and low birth weight (%) for 640 districts of India

Figure S2: Sample distribution of the excluded participates in the analyses of birth weight

Note: The excluded sample size is calculated by subtracting the valid sample of birth weight from the valid sample of small birth size. For the variable of small birth size, the information of all participants has been collected. Negligible participants (1.7%) have reported ‘don’t know’ which were excluded from the analyses.

*****************
